# Genome-wide identification and characterization of NBS-encoding genes in the sweet potato wild ancestor *Ipomoea trifida* (H.B.K.)

**DOI:** 10.1515/biol-2022-0052

**Published:** 2022-05-12

**Authors:** Zengzhi Si, Yake Qiao, Kai Zhang, Zhixin Ji, Jinling Han

**Affiliations:** Hebei Key Laboratory of Crop Stress Biology, Hebei Normal University of Science & Technology, Qinghuangdao, 066000, Hebei Province, China

**Keywords:** sweet potato, NBS-encoding genes, phylogenetic analysis, chromosomes location, expression profiles

## Abstract

The most predominant type of resistance (R) genes contain nucleotide-binding sites and leucine-rich repeat (NBS-LRR) domains, characterization of which is helpful for plant resistance improvement. However, the NBS genes of *Ipomoea trifida* (H.B.K.) remain insufficient to date. In this study, a genome-wide analysis of the NBS-encoding gene in *I. trifida* (H.B.K.) was carried out. A total of 442 NBS encoding genes were identified, amounting to 1.37% of the total genes of *I. trifida* (H.B.K.). Based on the analysis of the domains, the identified *ItfNBS* genes were further classified into seven groups: CNL, NL, CN, N, TNL, TN, and RNL. Phylogenetic analysis showed that the *I. trifida* NBS genes clustered into three independent clades: RNL, TNL, and CNL. Chromosome location analysis revealed that the distribution of *ItfNBS* genes in chromosomes was uneven, with a number ranging from 3 to 45. Multiple stress-related regulatory elements were detected in the promoters of the NBS-encoding genes, and their expression profiles were obtained. The qRT-PCR analysis revealed that *IbNBS10*, *IbNBS20*, *IbNBS258*, and *IbNBS*88 responded to stem nematode infection. These results provide critical proof for further characterization and analysis of NBS-encoding genes with important functions.

## 1 Introduction

Plants are often challenged by pathogens and pests during their lifetime [1]. In order to protect themselves, plants have formed an effective immune system in the process of evolution [1,2]. The immune system is divided into pattern-triggered immunity (PTI) and effector-triggered immunity (ETI) [3]. PTI responds to pathogen-associated molecular patterns through extracellular recognition receptors. It is considered to be the first line of defense and is easily attacked by evolving pathogens [4]. The second-layer immune system ETI perceives effectors directly or indirectly through the proteins encoded by disease resistance genes (R genes) [1]. Among these R genes, NBS-encoding genes are accounted for the largest proportion [5]. Approximately 80% of the characteristic R genes contain a central NBS domain and a C-terminal leucine-rich repeat (LRR) domain [6].

With the rapid development of next-generation sequencing technology, the NBS-encoding genes attracted more and more attention. Identification and characterization of the NBS gene family have been implemented in various species, such as *Arabidopsis thaliana* [7], *Oryza sativa* [8], *Glycine max* [9], *Lotus japonicas* [5], *Triticum aestivum* [10], *Arachis duranensis,* and *Arachis ipaensis* [11], and blueberry [12]. However, the information about NBS-encoding genes in *I. trifida* (H.B.K.) remains largely unknown.

*Ipomoea*, which consists of 600–700 species, is the largest genus in the family Convolvulaceae [13]. Of these species, sweet potato is the only species widely planted as a crop all around the world. Compared with its importance, the achievements in genomics and genetics of sweet potato were limited due to its complex genome nature [14]. A haplotype-resolved genome assembly of *Ipomoea batatas* was recently released and provided helpful information for studying the sweet potato genome [15]. Based on these reference genome sequences, a total of 315 NBS-encoding genes were identified with sweet potato recently [16]. Considering that the assembly of the sweet potato genome is still in progress [17], the number of NBS-encoding genes identified with the sweet potato might be underestimated.

*I. trifida* (H.B.K.) belongs to the family Convolvulaceae. It is one of the closest wild relatives of sweet potato [18] and most likely the diploid ancestor of the hexaploid sweet potato [13]. Compared to sweet potato, diploid *I. trifida* (H.B.K.) is more resistant to biological and abiotic stresses [19] and is an important wild species for evolutionary analysis and genetic improvement of sweet potato. Owing to its smaller genome size, diploid *I. trifida* (H.B.K.) is an ideal model species for studying the genome of sweet potato.

In 2015, an assembly of *I. trifida* was released [13], giving us the first insight into its genome. In 2018, a higher quality diploid *I. trifida* genome assembly was deposited into GenBank BioProject [17]. Based on the latter genome assembly, a genome-wide analysis of the NBS-encoding gene family of *I. trifida* has been implemented in the present study. The phylogenetic and mapping information were provided. The gene duplication and intron/exon distribution were analyzed. The evolution history, stress-related regulatory elements, and expression information were exposited. These results are hoped to provide insight into the evolution of NBS-encoding genes in the genome of *I. trifida*. This work provides fundamental information for further functional studies of *ItfNBSs* and the future molecular breeding of sweet potatoes.

## 2 Materials and methods

### 2.1 Data used in this study

The genome sequences of *I. trifida* including the predicted gene model annotation were downloaded from GenBank BioProject (accessions PRJNA428214) on December 15, 2018.

### 2.2 Identification of NBS-encoding genes from *I. trifida*

To identify NBS-encoding genes from the genome of *I. trifida*, both BLAST searches and hidden Markov model search (HMMsearch) were performed as described previously [20]. The protein sequences were first searched for the NB-ARC domain (Pfam accession number: PF00931) using HMMsearch with default parameters. Meanwhile, the reference sequences of NB-ARC (PF00931) were used to search for all protein sequences in *I. trifida* genome using the all-vs-all method. To filter out most of the spurious hits, the threshold expectation value was set to 1 × 10^−10^. After that, the genes gained by hidden Markov model (HMMsearch) and BLAST methods were merged, and the redundant ones were removed. To confirm whether the remaining sequences indeed contained the NB-ARC domain, they were subjected to online Pfam for further analysis (http://pfam.xfam.org/) (*E*-value up to 0.0001) [20, 21]. Sequences lacking NB-ARC domain were then removed. To determine whether the candidate NBS-encoding genes encoded TIR, RPW8, or LRR motifs, the Pfam database (http://pfam.xfam.org/), SMART protein motif analyses (http://smart.embl-heidelberg.de/), and Multiple Expectation Maximization for Motif Elicitation (MEME) were used. The COILS program was also conducted to detect potential CC motifs in the NBS-encoding genes, with a threshold value of 0.9 [22].

### 2.3 NBS-associated conserved domains

To identify additional domains such as TIR, CC, or RPW8 in the N-terminal domain and a variable number of LRR domains in the carboxy-terminal region, the raw TIR HMMs (PF01582), RPW8 (PF05659), and LRR (PF00560, PF07723, PF07725, PF12799, PF13306, PF13516, PF13855, and PF14580) were downloaded (http://pfam.xfam.org), so as to mine distinct domains in the identified NBS genes by using HMMsearch.

To investigate the structural motif diversity of the identified NBS genes, the protein sequences were subjected to motif analysis using MEME SUITE [23]. The criterion used for MEME analysis was as follows: (1) minimum width was 6; (2) maximum width was 50; (3) the maximum number of motifs was designed to identify 20 motifs; and (4) the iterative cycles were set by default.

### 2.4 Sequence alignment and phylogenetic analysis

The NBS domains (Ploop to MHDV) of the *I. trifida* NBS genes were aligned using Clustal Omega [24,25]. Genes with short NBS domains or divergent NBS domains were eliminated from the matrix using MEGA 7.0 [26], since these interfered with fine alignment and phylogenetic analysis [27,28]. According to the methods of Liu et al. (2021) [28], phylogenetic analyses were performed using IQ-TREE with the maximum likelihood algorithm [29], ModelFinder was used to estimate the best-fit model of nucleotide substitution [30], branch to support values were calculated using SH-aLRT [31], and UFBoot2 [32] with 1000 bootstrap replicates, and *Streptomyces coelicolor* accession P25941 as an outgroup [7]. Thus, the obtained tree was summated to Figtree for visual enhancement [33].

### 2.5 Chromosomal distribution and gene duplication

Relevant information on all identified *I. trifida* NBS genes were gained from the Sweet potato Genomics Resource (http://sweet potato.plantbiology.msu.edu/gt4sp_download.shtml). The NBS genes with chromosome located position were mapped on the chromosomes of *I. trifida* using MapChart (ver.2.30) software [34].

To search for potential duplicated NBS-LRRs in *I. trifida*, MCScanX software (http://chibba.pgml.uga.edu/mcscan2/) was used [35]. All the NBS protein sequences of *I. trifida* were compared to themselves by using the BLASTP program with an *E*-value of 1 × 10^−10^. The resulting blast hits and chromosome coordinates of all NBS-encoding genes were incorporated as input for MCScanX analysis. The hits were classified into various duplications, including segmental, tandem, proximal, and dispersed under the default criterion. The final results were drawn using CIRCOS software for visualization [36].

The aligned protein sequences of the *I. trifida* NBS-encoding genes were first converted into the corresponding nucleotide sequences using PAL2NAL software [37] and then summited to PAML 4.0 software [38] for *K*_*a*_/*K*_*s*_ (nonsynonymous/synonymous) calculation. According to the formula *T* = *K*_*s*_/2*r* (*r* = 6.5 × 10^−9^) [39], the reproduction period of different gene pairs was also calculated.

### 2.6 Identification and analysis of the promoter regions

The 1,500 bp promoter sequences of *I. trifida* genes were obtained from the genome sequence of *I. trifida*. PLANT CARE (http://bioinformatics.psb.ugent.be/webtools/plantcare/html/) was used to determine the cis-acting regulatory elements and to analyze the promoter sequences of *I. trifida* NBS genes.

### 2.7 Expression analysis of NBS-encoding genes in *I. trifida*

The expression profiles were downloaded from the Sweet potato Genomics Resource (http://sweetpotato.plantbiology.msu.edu). A heat map was produced to distribute the expression levels using the RPKM (i.e., reads per kilobase per million) value in MeV software [40].

### 2.8 RNA isolation and qRT-PCR analysis

The storage roots of JK20 (a strain saved in our laboratory and resistant to sweet potato stem nematodes, data unpublished) were inoculated with stem nematodes according to the previously reported methods [41]. Samples (whole storage roots) were collected at eight time points: 0 h, 6 h, 12 h, 1, 2, 4, and 6 days after stem nematode infection. Thereafter, the total RNA of the samples was isolated using RNAprep Pure Plant Kit (Tiangen Biotech, Beijing, China), and the first-strand cDNA was prepared using Quantscript Reverse Transcriptase Kit (Tiangen Biotech). The sweet potato *β-actin* gene (Genbank AY905538) was selected and used to normalize the relative quantities of the target genes based on its consistency across the different time points [42]. Three replications were performed, and the expression changes were calculated using the 2^–ΔΔCt^ method for each sample [43]. Then, quantitative real-time polymerase chain reaction (qRT-PCR) was performed as described previously [44]. The primers used for PCR were designed using Primer-BLAST software [45] (Table S1).

## 3 Results

### 3.1 Identification and classification of the NBS genes in *I. trifida*

A total of 442 NBS-encoding protein sequences were identified with the genome of *I. trifida* and were further classified as different groups (Table 1 and Table S2). Of them, 175 candidates with full-length domains of CC, NBS, and LRR were denoted as CNL; 35 candidates containing complete domains of TIR, NBS, and LRR were grouped as TNL; and 2 candidates with complete RPW8, NBS, and LRR domains were classed as RNL. Then, 58 candidates containing the CC and NBS domains were typed as CN, 8 candidates containing the TIR and NBS domains were typed as TN, and 118 candidates containing the NBS and LRR domains were typed as NL. The other 46 candidates only containing the NBS domain were typed as N. The most abundant type was CNL, followed by NL, CN, N, TNL, TN, and RNL.

**Table 1:** Compare analysis of NBS-encoding genes in the genomes of *I. trifida* and *I. batatas*

Protein domains	Letter code	*I. trifida*		*I. batatas*	
		Number	Percentage	Number	Percentage
CC-NBS-LRR	CNL	175	39.59	92	35.38
CC-NBS	CN	58	13.12	12	4.62
TIR-NBS-LRR	TNL	35	7.92	41	15.77
TIR-NBS	TN	8	1.81	0	0.00
CC-RPW8-NBS-LRR	CRNL	0	0.00	7	2.69
RPW8-NBS-LRR	RNL	2	0.45	0	0.00
NBS-LRR	NL	118	26.70	101	38.85
NBS	N	46	10.41	7	2.69
Others		0	0.00	0	0.00
Total NBS-encoding genes		442	100.00	260	100.00

^*^Source: *I. batatas* ([16]).

Among the 442 NBS genes, the shortest (*itf07g09670.t1*) was of 238 amino acids, while the longest (*itf07g02520.t1*) was of 3,145 amino acids, and the average length of the *ItfNBS* genes was 904.78 amino acids (Table S3). Of different types of *I. trifida* NBS genes, the average length of N-type NBS genes was the shortest (547.61 aa), followed by TN (644.88 aa), CN (737.76 aa), RNL (832.00 aa), NL (870.23 aa), CNL (976.23 aa), and TNL (1473.71 aa) (Table S4). The average number of exons among the NBS genes in the *I. trifida* genome was 3.80, with a range from 1 to 28. The average number of TNL-type NBS genes was the largest (10.80), RNL was the next (5.00), while the other types of NBS genes were nearly the same (Table S4).

As shown in Table 1, the total number of NBS-encoding genes identified with the *I. trifida* and sweet potato genome was 442 and 260, respectively. In this study, the NBS-encoding genes identified with *the I. trifida genome were more than those in the sweet potato genome*. Among different types of NBS-encoding genes, the number of CNLs in the genome of *I. trifida* and sweet potato was larger than that of TNLs. The previous study showed that the seven CRNLs identified with sweet potato contained RPW8, NBS, LRR domain, as well as CC domain. However, the two RNLs identified with *I. trifida* contained RPW8, NBS, and LRR domain, but not CC domain.

### 3.2 Analysis of the conserved motifs of the NBS genes

**Figure 1:**
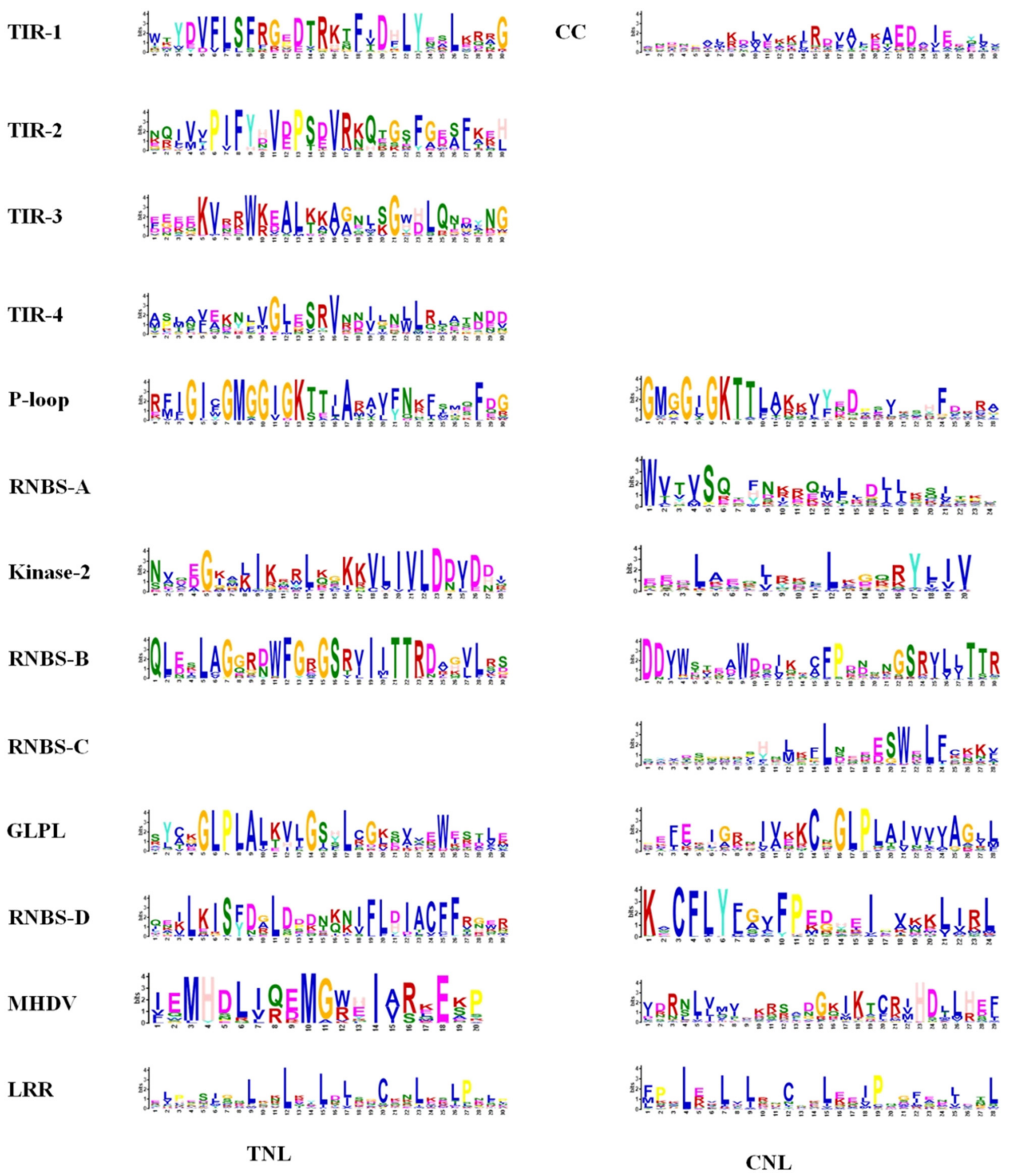
MEME analysis of the TNL and CNL proteins in *I. trifida*. Different colored letters represent amino acids belonging to the different families.

According to the above-mentioned analysis, a total of 175 CNL-type genes and 35 TNL-type genes with complete structures were selected for subsequent analysis. The MEME results showed that the P-loop, kinase-2, RNBS-B, RNBS-D, GLPL, MHDV, and LRR motifs were detected in both CNL and TNL members, and the P-loop, kinase-2, GLPL. Of them, MHDV displayed a high similarity between the two types of proteins, while the RNBS-D motif presented low similarity between them (Figure 1, Figures S1 and S2). The CC, NBS-A, and -C motifs were detected in the CNL-type genes while absent in TNL-type genes (Figure 1, Figures S1 and S2). As expected, the TIR-1, TIR-2, TIR-3, and TIR-4 were detected among TNL-type genes while absent from CNL-type genes (Figure 1, Figures S1 and S2). Moreover, the MHDV motif in the CNL and TNL proteins was modified into an MHDL motif (the consensus sequence was not MHDV but MHDL) (Figure 1, Figures S1 and S2).

### 3.3 Phylogenetic analysis

**Figure 2:**
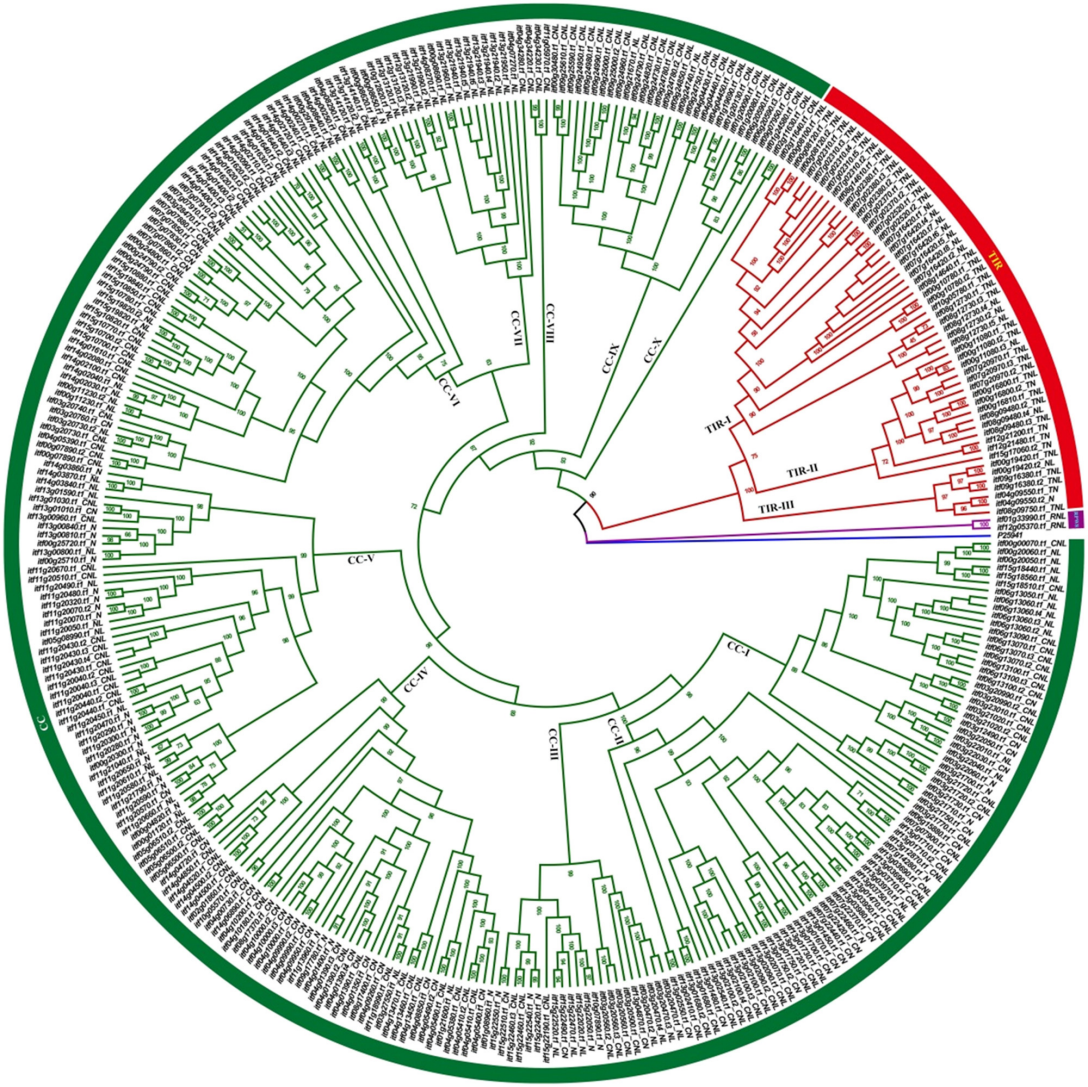
Phylogenetic reconstruction of the NBS-LRR proteins in *I. trifida*. A tree was constructed using NBS domains. Bootstrap values (1,000 iterations) are indicated in every branch. Each protein is encoded as follows: Gene ID + Domains present (TNL, CNL, RNL, CN, TN, NL, and N). Purple, red, and green correspond to RNL, TNL, and CNL groups, respectively. P25941 was used as an outgroup and was marked as blue.

In order to analyze the evolutionary relationships of the *I. trifida* NBS genes, a phylogenetic tree was constructed using the conserved NBS domains. After removing sequences of incomplete and divergent NBS domains, 355 sequences were left to use. As shown in Figure 2, the *I. trifida* NBS genes clustered into three independent clades: RPW8, TIR, and CC. Two genes (*itf01g33990.t1* and *itf12g05370.t1*) contained RPW8 domains were clustered in the PRW8 clade; 54 genes, including all of the TNL- and TN-type genes and part of NL- and N-type genes, were clustered in the TIR clade; while the remaining 299 genes, including all of the CNL- and CN-type genes and part of NL- and N-type genes, were clustered in the CC clade (Figure 2). The TIR clade was further classed into three subgroups: TIR-I, TIR-II, and TIR-III, with the TIR-I containing the largest number of genes. The CC clade was further classed as ten subgroups: CC-I, CC-II, CC-III, CC-IV, CC-V, CC-VI, CC-VII, CC-VIII, CC-IX, and CC-X, and the number of genes in CC-I, CC-II, CC-IV, CC-V, CC-VI, and CC-IX was larger than that in other subgroups (Figure 2).

**Figure 3:**
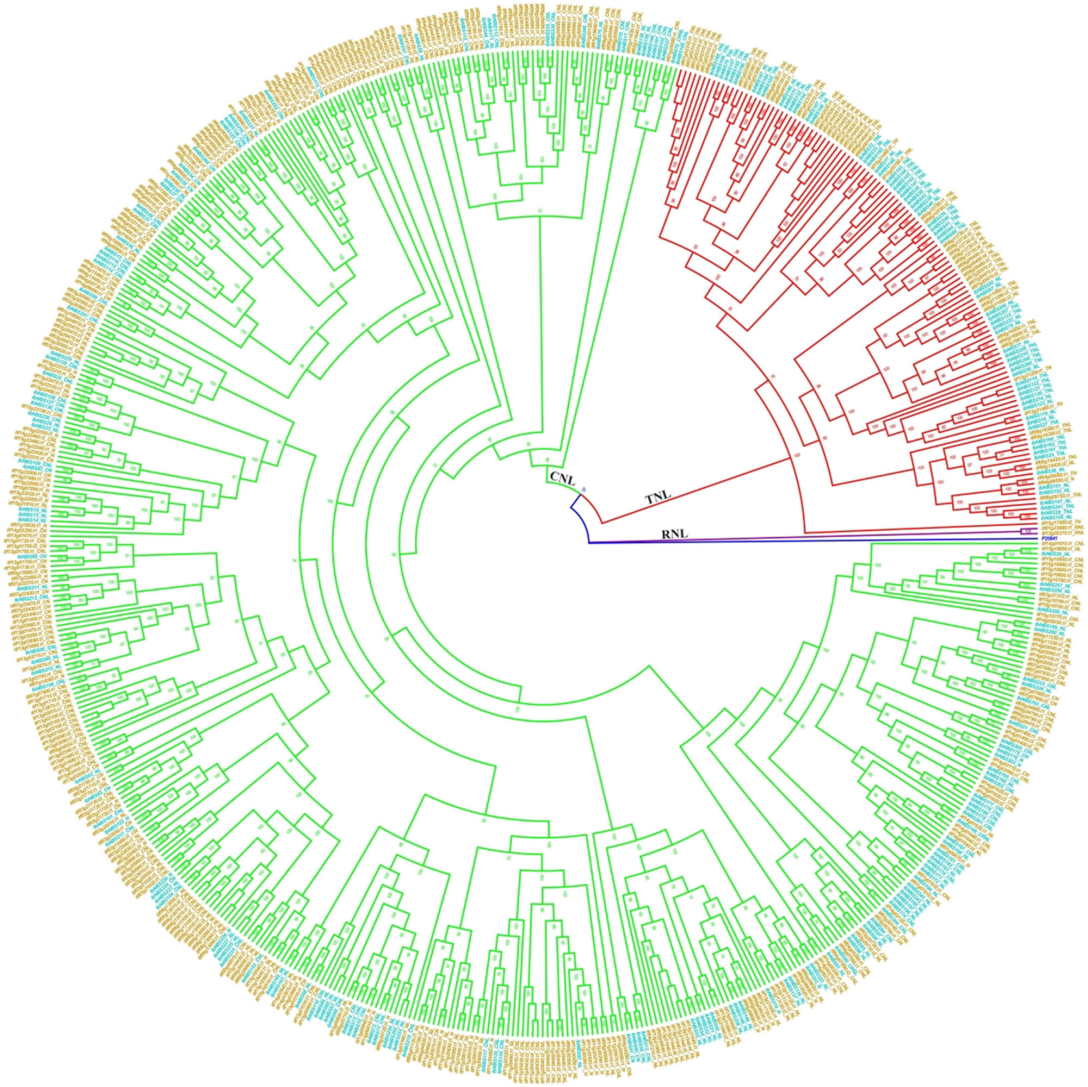
Phylogenetic tree of NBS-encoding genes from *I. trifida* and sweet potato. Each protein is encoded as follows: Gene ID + Domains present (TNL, CNL, RNL, CN, TN, NL, and N). The proteins of different species were separated by the color of the names: auqamarin (*I. trifida*) and cyan (sweet potato). Purple, red, and green clades correspond to RNL, TNL, and CNL groups, respectively. P25941 was used as an outgroup and was marked as blue.

For comparative purposes, a second phylogenetic tree, including NBS-encoding genes of *I. trifida* and sweet potato, was constructed (Figure 3). Most of the clades were grouped as previously observed. With P25941 as an outgroup, the NBS-encoding genes of the two species formed three independent clades: RNL, TNL, and CNL (Figure 3). All of the genes containing the TIR domain were grouped into the TIR clade; the genes containing the CC domain were grouped into the CNL clade; the genes in *I. trifida* containing the RPW8 domain formed the RNL clade, while the ones in sweet potato (CRNLs) grouped in the CNL clade.

### 3.4 Chromosomal distribution of *I. trifida* NBS-encoding genes

**Figure 4:**
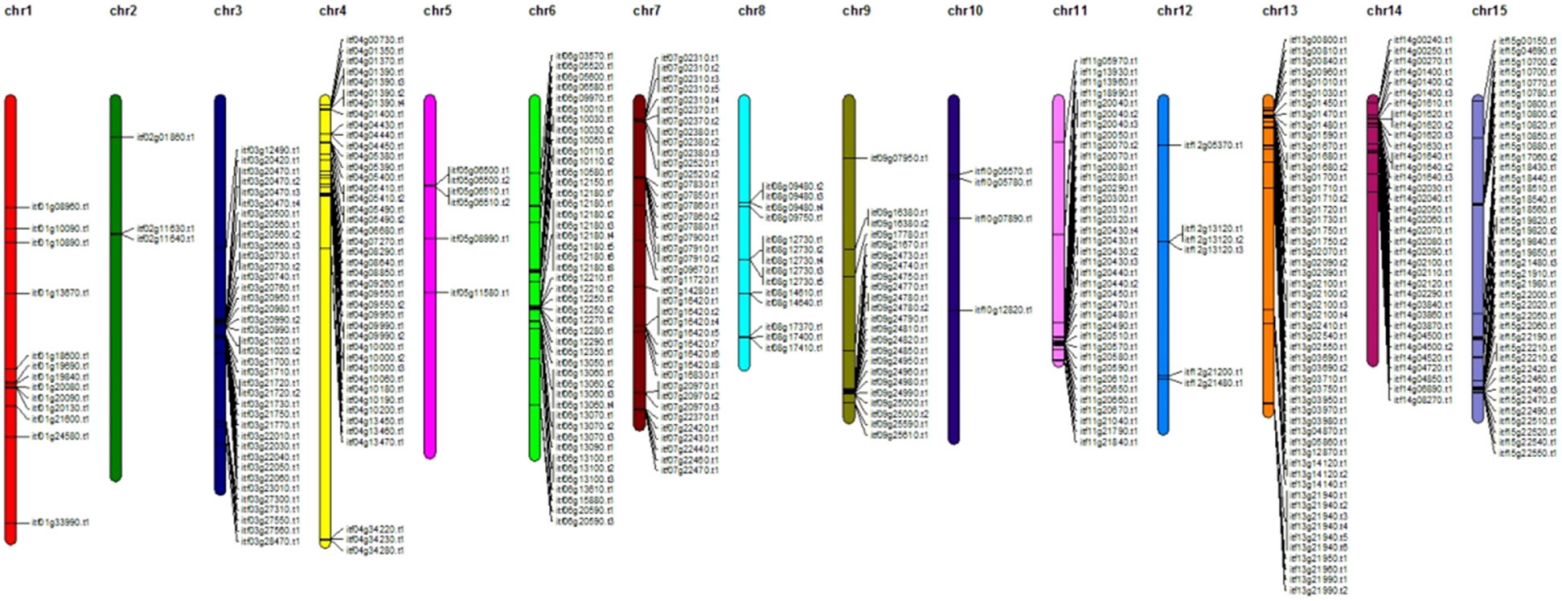
Physical location of *I. trifida* NBS-encoding genes on chromosomes. Chromosome numbers were indicated above each chromosome. Chromosome size is indicated by the relative length.

In the genome of *I. trifida*, 43 NBS-encoding genes that could not be mapped on the 15 chromosomes were discarded. Among 442 *I. trifida* NBS genes reported in this study, 399 were assigned to their chromosomal locations based on their comparison with the physical map of *I. trifida* (Figure 4). The remaining 43 genes, which could not be mapped for lacking enough genome information, were not analyzed. As shown in Figure 4, the chromosomal distribution of the NBS-encoding genes is non-random: 53, 45, 42, 40, and 40 of them were located on chromosomes 13, 6, 4, 7, and 15, respectively; 38, 37, 35, 23, 14, and 13 of them were located on chromosomes 3, 11, 14, 9, 8, and 1, respectively, while only 6, 6, 4, and 3 of them were located on chromosomes 5, 12, 10, and 2, respectively. No noticeable difference was found between the distributions of the TNL and CNL types of genes on the chromosomes (Figure 4).

### 3.5 Duplication pattern and adaptive evolution analysis of ItfNBS family in *I. trifida* genome

**Figure 5:**
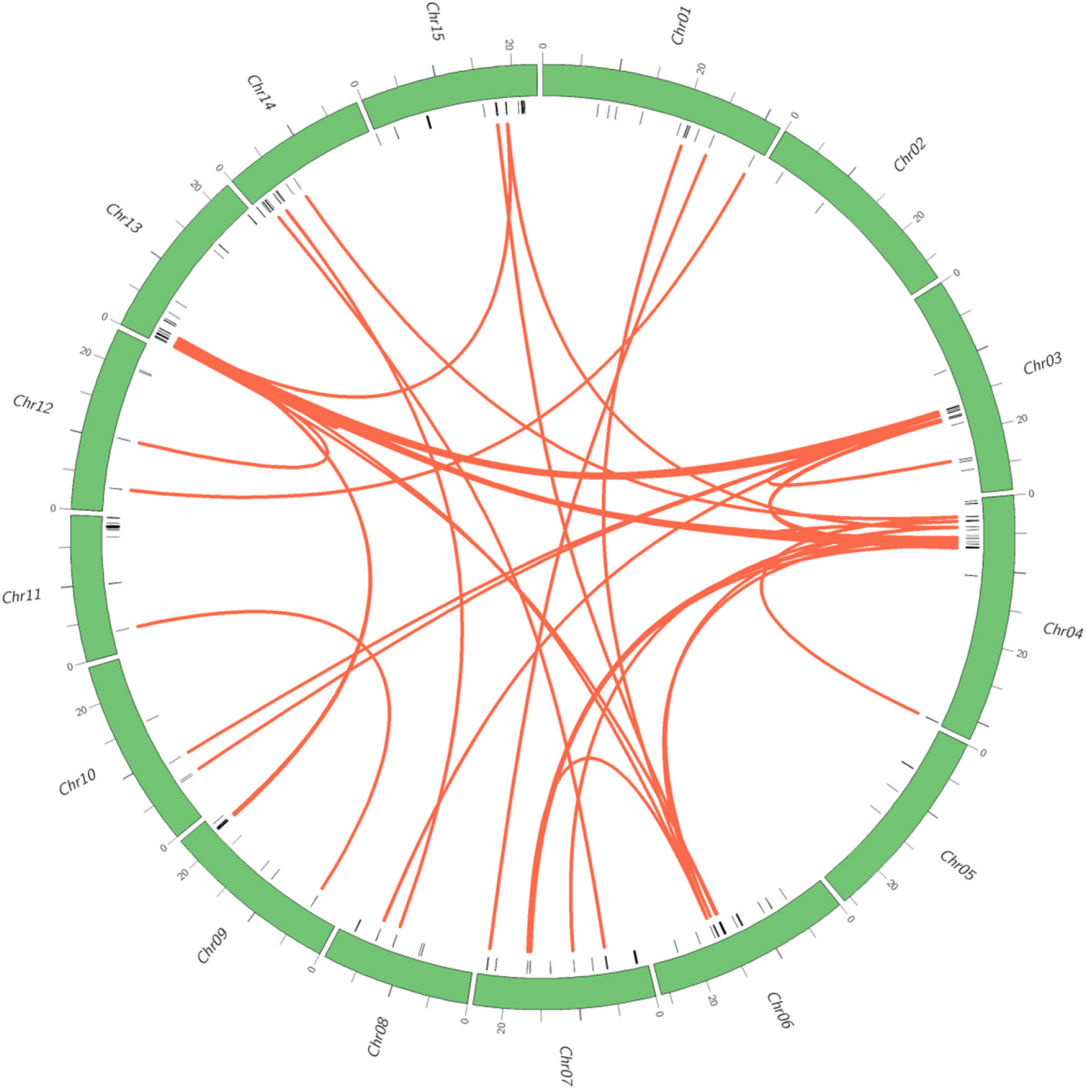
Collinear gene pairs for *I. trifida* NBS genes on 15 chromosomes. The out circle represents the haploid chromosomes of *I. trifida* (green); the second circle represents the matches of the NBS-encoding genes with the genome of *I. trifida* (black). Red lines show the collinear gene pairs for *I. trifida* NBS-encoding genes.

To study the gene duplication events of *ItfNBS* family, the duplication events of NBS-encoding genes in *I. trifida* were analyzed by using MCScanX software. Of the 399 NBS encoding genes, 32 were classed as dispersed, 16 as proximal, 273 as tandem, and 78 as segmentally duplicated (Figure 5). All of the 273 tandem gene pairs were located on the same chromosomes; 59 of the 78 segmentally duplicated genes formed 50 pairs of collinear NBS encoding genes. As shown in Figure 5, the detected collinear genes were distributed among all of the chromosomes except chromosomes 2 and 5, and chromosome 13 formed the most collinear genes (Figure 5). Five pairs of the detected collinear genes were formed by both of the genes located in the same chromosome; the other pairs were formed by two genes from different chromosomes.

The *K*_*a*_/*K*_*s*_ ratios of the duplicated ItfNBS gene pair were calculated for adaptive evolution analysis. As shown in Table S5, 43 of the homologous gene pairs had <1 *K*_*a*_/*K*_*s*_ ratios (ranging from 0.001 to 0.99), suggesting these genes probably underwent purifying selection. The *K*_*a*_/*K*_*s*_ ratios of other 29 homologous genes pairs were with >1 *K*_*a*_/*K*_*s*_ ratios, and the *K*_*a*_/*K*_s_ ratios of *itf13g02100.t3_itf13g02100.t2* (9.18) and *itf07g16420.t7_ itf07g16420.t6* (36.24) were all immensely larger than 1, indicating that these genes probably underwent positive selection. The replication time analysis of these homologous genes showed that the duplication of the investigated gene pairs probably occurred between 0.0014 and 97.07 MYA (million years ago) (Table S5).

### 3.6 Stress-related regulatory elements in the putative promoters of *I. trifida* NBS-encoding genes

The 1,500 bp upstream regulatory regions of all the 442 *I. trifida* NBS encoding genes were used to explore stress-related regulatory elements. After removing NBS encoding genes for lack of upstream genome sequences, the remaining 227 genes were further analyzed. Of them, the most abundant elements related to stress were CGTCA-motif, followed by ABRE, MBS, TGA-element, LTR, TC-rich repeats, WUN-motif, and MBSI (Figure S3). Of the 227 *I. trifida* NBS-encoding genes, the number of stress-related regulatory elements was different, more (9) were predicted in 13 *ItfNBS* genes (*itf00g16800.t1*, *itf00g16800.t2*, *itf04g01390.t1*, *itf04g01390.t2*, *itf06g15880.t1*, *itf11g20430.t1*, *itf11g20430.t2*, *itf11g20430.t3*, *itf11g20430.t4*, *itf13g02100.t1*, *itf13g02100.t2*, *itf13g02100.t4,* and *itf14g00250.t1*), while only one was predicted in 21 *ItfNBS* genes (*itf00g08100.t1*, *itf03g28470.t1*, *itf04g05490.t2*, *itf04g10180.t1*, *itf06g12180.t1*, *itf06g12180.t2*, *itf06g12180.t5*, *itf06g12180.t6*, *itf06g12210.t1*, *itf06g12210.t2*, *itf06g13090.t1*, *itf06g13100.t1*, *itf06g13100.t2*, *itf06g13100.t3*, *itf07g02520.t1*, *itf07g02520.t2*, *itf13g03690.t1*, *itf13g03690.t2*, *itf15g10800.t1*, *itf15g10850.t1*, and *itf15g19850.t1*) (Figure S3).

### 3.7 Expression patterns of NBS-encoding genes in *I. trifida*

**Figure 6:**
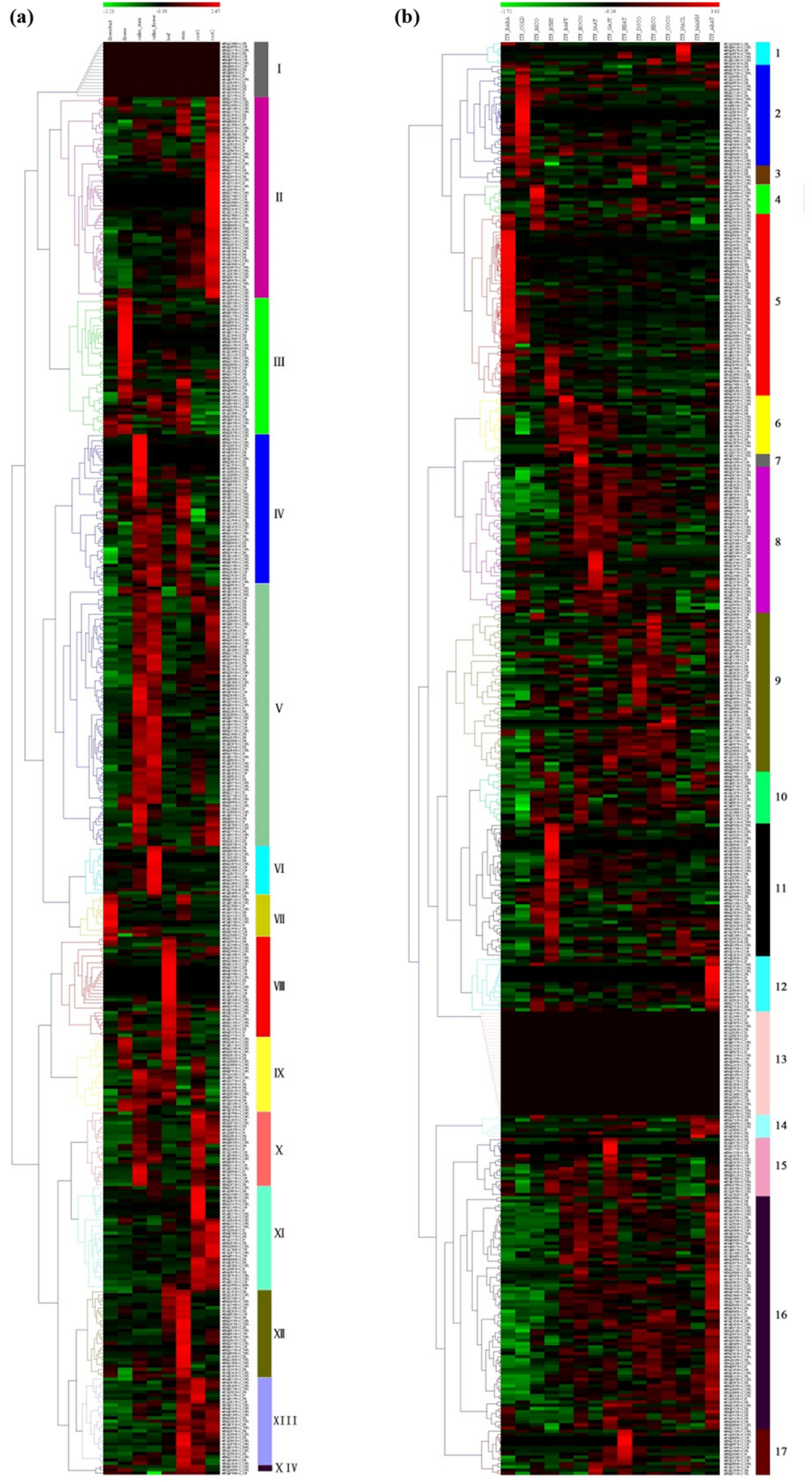
Heatmap of expression profiles for *I. trifida* NBS genes. (a) Expression profiles of NBS-encoding genes in different *I. trifida* tissues; (b) expression profiles of *I. trifida* NBS-encoding genes under different stress.

To analyze the expression patterns of NBS-encoding genes in *I. trifida*, the raw RPKM values of gene expression profiles were downloaded, and two heat maps were generated (Figure 6). As shown in Figure 6, the left heat map showed the expression of *I. trifida* tissues: flower bud, flower, callus stem, callus flower, leaf, stem, root1, and root2; the right one displayed the expression of *I. trifida* abiotic and biotic stress treatments: beta-aminobutyric acid biotic stress experiment (ITF_BABA), cold stress at 10/4℃ day/night experiment (ITF_COLD), biotic stress control (ITF_BICO), benzothiadiazole S-methylester biotic stress experiment (ITF_BTHT), 6-benzylaminopurine 10 µM hormone stress experiment (ITF_BAPT), hormone control experiment (ITF_HOCO), indole-3-acetic acid 10 µM hormone stress experiment (ITF_IAAT), gibberellic acid 50 µM hormone stress experiment (ITF_GA3T), heat stress at 35/35℃ day/night experiment (ITF_HEAT), drought and salt control experiment (ITF_DSCO), heat control at 28/22℃ day/night experiment (ITF_HECO), cold control at 28/22℃ day/night experiment (ITF_COCO), NaCl salt stress experiment (ITF_NACL), mannitol drought stress experiment (ITF_MANN), and abscisic acid 50 µM hormone stress experiment (ITF_ABAT) (Figure 6).

Fourteen clusters of expression patterns were observed in the *I. trifida* tissues. Cluster V contained the largest number of genes (81), followed by cluster II (62), cluster IV (46), cluster III (42), cluster XI (32), cluster VIII (31), cluster XII (27), cluster XIII (27), cluster IX (23), cluster X (23), cluster I (17), cluster VI (15), cluster VII (13), and cluster XIV (3). Cluster I consisted of 17 genes, and none of them were expressed in either of the observed tissues. The genes contained in clusters IV and V were mainly upregulated in callus stem and callus follower while downregulated in flower buds, follower, and leaf; more than a half of the genes in cluster IV were also upregulated in the stem, while the genes in cluster V were mainly downregulated in this tissue. Nearly all genes in cluster II, and more than half of the ones in cluster XI were upregulated in root2, and almost all of the genes in cluster XI were upregulated in root1. The genes in cluster III were mainly upregulated in followers, while the genes in clusters VII, VIII, IX, X, XII, XIII, and XIV were mainly upregulated in flower bud, leaf, leaf, root1, stem, stem, and stem, respectively. The genes in cluster XIII and cluster XIV were also detected upregulated in root1, nearly a half of the ones in cluster XIII were upregulated in root2, and almost all of them were downregulated in follower bud and flower.

Seventeen clusters of expression patterns were observed in the *I. trifida* stress treatments. Cluster 16 contained the largest number of genes (72), followed by cluster 5 (56), cluster 9 (49), cluster 8 (45), cluster 11 (41), cluster 13 (32), cluster 2 (31), cluster 6 (18), cluster 15 (18), cluster 12 (17), cluster 10 (16), cluster 17 (14), cluster 4 (9), cluster 1 (7), cluster 14 (7), cluster 3 (6), and cluster 7 (4). Cluster 13 consisted of 31 genes, and none of them was expressed in either of the stress treatments. In the beta-aminobutyric acid biotic stress experiment (ITF_BABA), the genes in cluster 5 were mainly upregulated, while in clusters 8, 10, and 16 were mainly downregulated. In the cold stress at 10/4℃ day/night experiment (ITF_COLD), the genes in cluster 2 were mainly upregulated, while downregulated in the biotic stress control (ITF_BICO), the genes in cluster 4 were mainly upregulated. In the benzothiadiazole S-methylester biotic stress experiment (ITF_BTHT), the genes in cluster 11 were mainly upregulated; the genes in cluster 16 were mainly downregulated. The genes in cluster 1 were mainly upregulated in the NaCl salt stress experiment, while the genes in clusters 3 and 4 were mainly upregulated in the drought and salt control experiment (ITF_DSCO). The genes in clusters 6, 7, and 8 were mainly upregulated in the hormone control experiment (ITF_HOCO), indole-3-acetic acid 10 µM hormone stress experiment (ITF_IAAT), and gibberellic acid 50 µM hormone stress experiment (ITF_GA3T). The genes in cluster 16 were upregulated in the abscisic acid 50 µM hormone stress experiment while downregulated in the beta-aminobutyric acid biotic stresses experiment (ITF_BABA) and 10/4℃ day/night experiment (ITF_COLD). The genes in cluster 17 were mainly upregulated in heat stress at 35/35℃ day/night experiments (ITF_HEAT).

### 3.8 qRT-PCR analysis of the NBS-encoding genes under treatments

**Figure 7:**
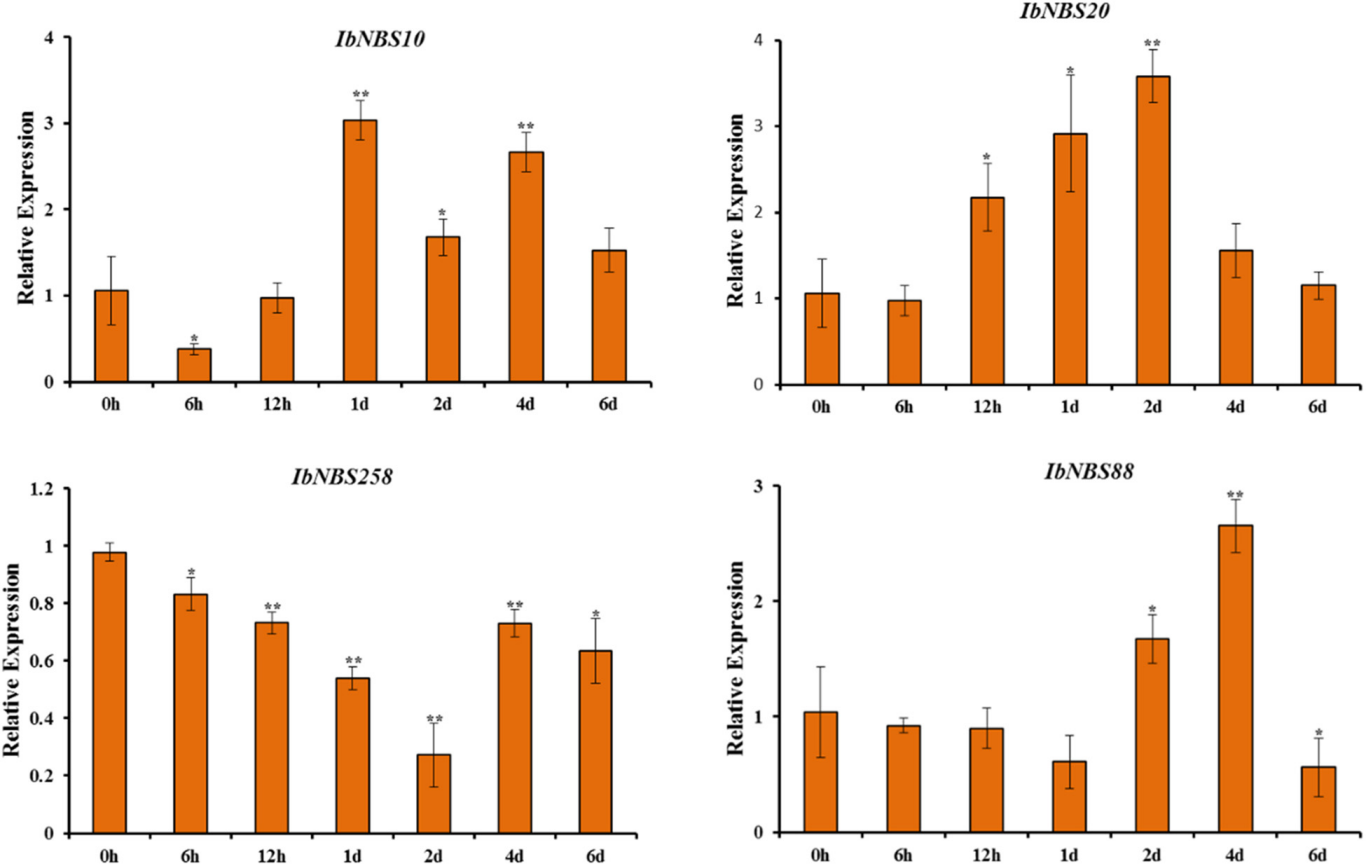
Expression analysis of *IbNBS10*, *IbNBS20*, *IbNBS258*, and *IbNBS*88 in storage roots of JK20 after different times of stem nematode inoculation.

According to the above results, three NBS genes of *I. trifida* (*itf06g20590.t3*, *itf13g01730.t1*, and *itf15g10880.t1*) from cluster 5 and two NBS genes of *I. trifida* (*itf15g10780.t1* and *itf06g20590.t1*) from cluster 16 were selected for further analysis, since these genes were potentially involved in biotic stress. Based on phylogenetic analysis and the results of BLAST, six NBS-encoding genes (*IbNBS10*, *IbNBS20*, *IbNBS258*, and *IbNBS*88, *IbNBS*246, and *IbNBS*212) of sweet potato were selected for primers designed, and four of them (*IbNBS10*, *IbNBS20*, *IbNBS258*, and *IbNBS*88) were successfully used in the subsequent qRT-PCR program (Figure 7).

Compared with the control conditions (0 h), *IbNBS10*, *IbNBS20*, *IbNBS258*, and *IbNBS*88 were all responding to stem nematodes infection. The transcripts of *IbNBS10*, *IbNBS20*, and *IbNBS88* were increased after stem nematode infection, while that of *IbNBS258* was decreased after treatment. The transcript of *IbNBS10* first peaked after 1-day stem nematode infection and second after 4-day treatment, with 3.03- and 2.67-fold higher expression levels than that of control. The transcript of *IbNBS20* peaked at 2d with a 3.58-fold higher expression level than that of control, and then, the expression level declined. The *IbNBS88* transcript peaked at 4d with a 2.66-fold higher expression level than that of control, and then, the expression level declined. The transcript of *IbNBS258* was decreased after 6 h stem nematode infections, dropped after 2-day treatment, and then increased to comparable normal expression after 6-day cold treatment.

## 4 Discussion

*I. trifida* (H.B.K.) is the most likely diploid ancestor of sweet potato [13] and contains excellent genes that were important but lost in the long-term manually selection of sweet potato. It is an ideal model species for evolutionary analysis and genetic improvement of sweet potato. NBS-encoding gene family was the most predominant R-genes. Genome-wide identification and characterization of NBS-encoding genes in *I. trifida* will help disease and insect resistance breeding of sweet potatoes.

In this study, the NBS-encoding genes in *I. trifida* genome were identified and characterized. A total of 442 NBS-coding genes were obtained, representing 1.37% of the total number of coding sequences [17]. The total number of NBS-coding genes obtained in this study was comparable to the number of that recently released by Liu et al.: a total of 432 NBS-encoding genes, of the 398 were classified as CNL, 32 as TNL, and 2 as RNL [28]. The frequency of NBS sequences in the *I. trifida* genome is medium to high level compared to other species, in which it ranged from 0.17 to 1.76% [12]. Thus, it is unlikely that the number of NBS genes has been dramatically over- or underestimated.

According to the presence or absence of the related domains, 175, 35, 2, 58, 8, 118, and 46 of the NBS-coding genes were classed as CNL, TNL, RNL, CN, TN, NL, and N. In *I. trifida*, both the CNLs and the TNLs were detected, and the number of CNLs was much higher than that of TNL, which is generally consistent with the results observed in many other dicots, such as cassava [46], potato [47], tomato [47], pepper [47], common bean [48,49], and blueberry [12]. As reported in a previous study, in dicotyledonous plants, both TNLs and non-TNLs may occur, while in monocots are non-TNLs [50]. Phylogenetic analysis revealed that the *I. trifida* NBS genes clustered as three independent clades: RPW8, TIR, and CC (Figure 2). The TIR clade was further classed into three subgroups, and the CC clade was further classed as ten subgroups. All of these results suggested that TNL, RNL, and CNL genes may originate from different recent ancestors, and then, the CNL genes may have undergone a more divergent differentiation than the TNL and RNL genes.

We also found that the NBS-encoding genes of *I. trifida* were distributed over all the 15 chromosomes while their distribution on chromosomes was uneven: the number of genes located on different chromosomes ranged from 2 to 45. The uneven chromosomal distribution has also been observed in other species, such as Arabidopsis [7], chickpea [51], or blueberry [12]. The phenomenon that the NBS-LRR genes are present in clusters has been frequently observed, and it may contribute to the genetic variation and the rapid evolution [52].

NBS-encoding genes are frequently clustered in the genome as tandem and segmental duplication [7], and both of them played an important role in gene family expansion [53]. Thus, in this study, both tandem and segmental were analyzed to investigate the duplication events. Of the 442 NBS-encoding genes, 273 were detected as tandem and 78 as segmentally duplicated. The results showed that tandem duplication is the main mechanism in forming NBS-encoding paralogs, indicating that novel resistant functions of NBS-encoding genes may be produced from them [54].

In order to investigate whether the positive selection occurred after duplication of *I. trifida* NBS-encoding family, the *K*_*a*_/*K*_*s*_ values of 72 homologous genes were calculated. The previous report showed that *K*_*a*_/*K*_*s*_ <1, =1, and >1 indicate purifying, neutral, and positive selection, respectively [4,55]. According to this criterion, most of the *I. trifida* NBS-encoding duplicated genes underwent purifying selection, while a few ones underwent positive selection, such as *itf13g02100.t3*_*itf13g02100.t2* and *itf07g16420.t7*_*itf07g16420.t6*. These results suggested that *I. trifida* was of a quite high chance of producing new functional genes [53]. Further analysis of these homologous genes showed that the duplication of the investigated gene pairs occurred between 0.0014 and 97.07 MYA (million years ago).

Studying the cis-acting elements will help us to get insight into the promoter activity and improve our basic understanding of gene regulation [56,57]. To explore the stress-related regulatory elements in the putative promoters of *I. trifida* NBS-encoding genes, the 1,500 bp upstream sequences of the NBS-encoding genes were analyzed. The obvious enrichment of ABRE, MBS, TGA-element, LTR, TC-rich repeats, WUN-motif, and MBSI suggested that these genes played important roles in defense mechanisms [12].

In this article, the levels of the *I. trifida* NBS-encoding gene expression were acquired; 429 of 442 NBS encoding genes were supported by expression evidence in different tissues and exhibited somewhat tissue distinct expression patterns; 411 of the NBS-encoding genes were supported by expression evidence in different stress treatments. The NBS-encoding genes were further grouped into 14 and 17 clusters respectively according to the distinct expression patterns of tissues and stress treatments. Moreover, the genes of a specific type (i.e., TNL, RNL, CNL, and so on) were grouped into different clusters, suggesting they exhibited diverse expression patterns of members. These results suggest that the functional diversification of a particular type *I. trifida* NBS-encoding genes and similar phenomena have also been reported in other species [58,59]. Based on expression patterns, phylogenetic analysis and the results of BLAST, *IbNBS10*, *IbNBS20*, *IbNBS258*, and *IbNBS88* were selected for qRT-PCR analysis. The results showed that they respond to stem nematode infection.

## 5 Conclusion

In summary, a total of 442 encoding genes were identified with the *I. trifida* genome. Phylogeny and physical location information of these genes may help identify novel functional resistance genes. Duplication pattern and phylogenetic analysis may illuminate the recent evolution and dynamics of *ItfNBS* genes. The distribution of stress-related regulatory elements, functional annotation, tissues, and stress treatments expression profiles may favor cloning genes related to tissue development, stress resistance, or stress tolerance. In a word, our data will help to broaden our horizon in studying NBS-encoding genes of *I. trifida* and molecular breeding of sweet potatoes in the future.

## Supplementary Material

Supplementary Material
