# Supplementary material for: Genome-wide identification and characterization of NBS-encoding genes in the sweet potato wild ancestor Ipomoea trifida (H.B.K.)
Source: Open Life Sci. 2022 May 12;17(1):497–511. doi: 10.1515/biol-2022-0052 (PMC9102303; doi:10.1515/biol-2022-0052)
Supplement: Supplementary Material [file biol-2022-0052-sm.pdf]

Supplementary material

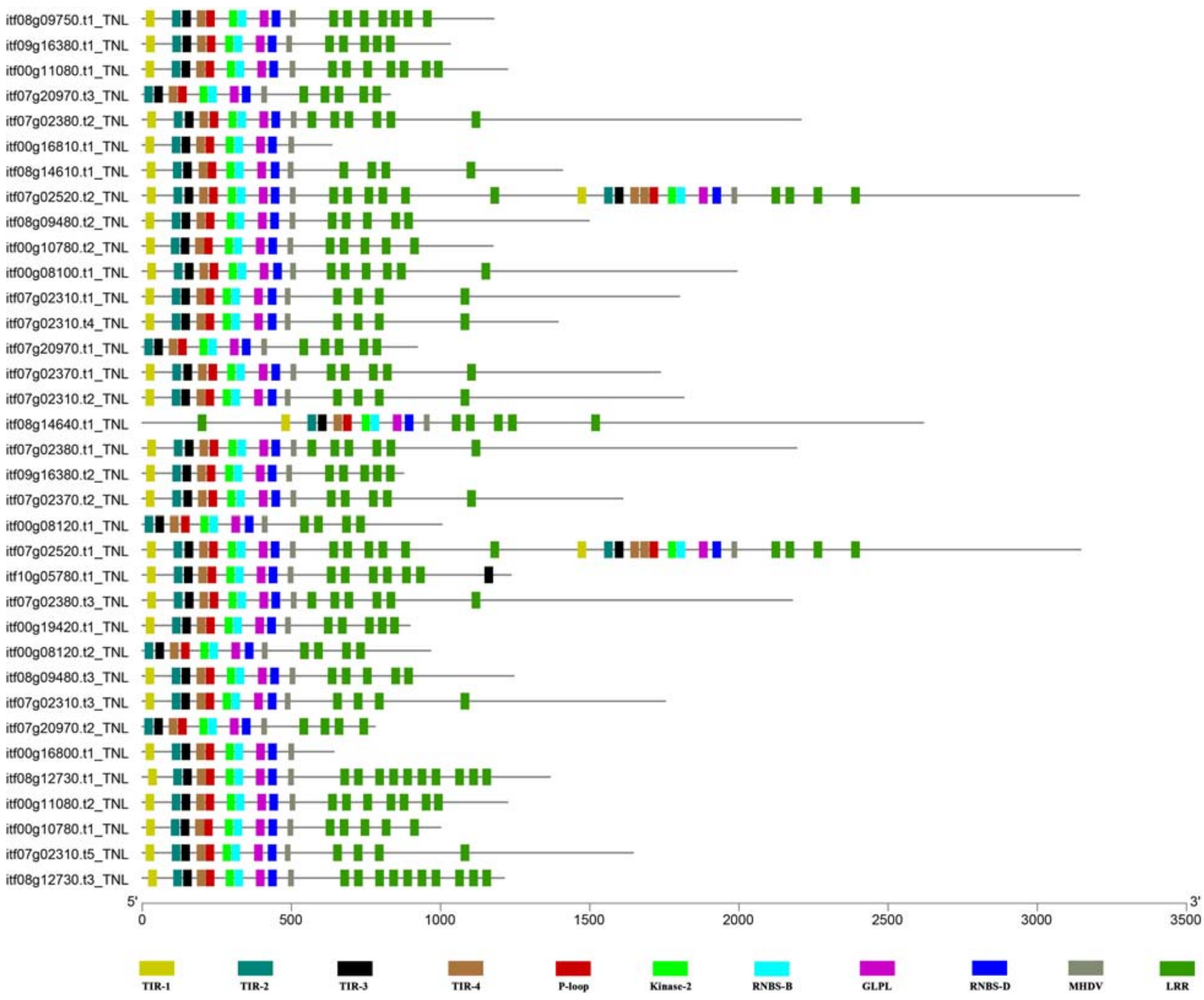

Figure S1: Composition of the conserved motifs in TIR-NBS-LRR genes in *I. trifida*.

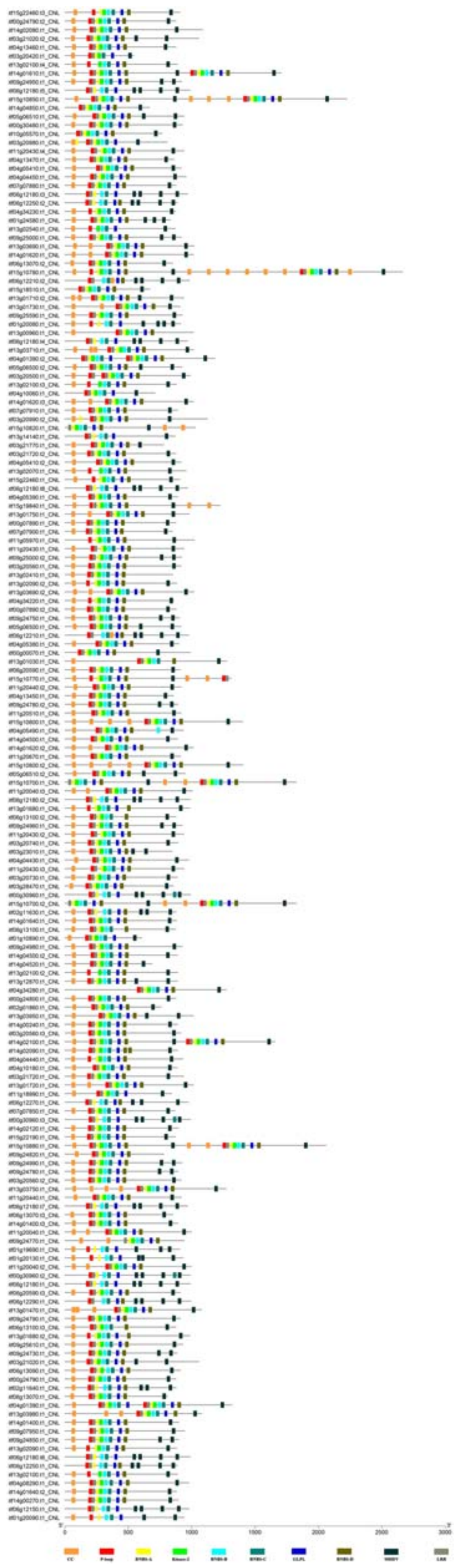

Figure S2: Composition of the conserved motifs in CC-NBS-LRR genes in *I. trifida*.

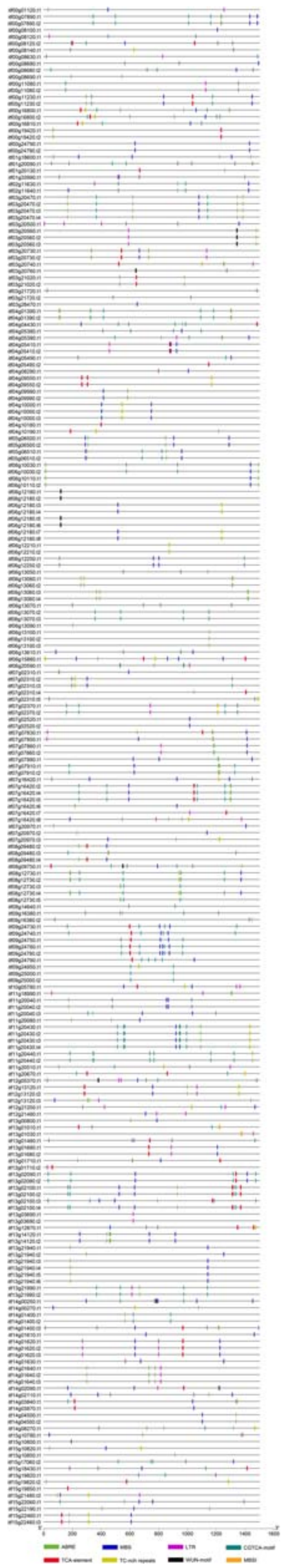

Figure S3: Prediction of cis-responsive elements in the 1500bp upstream regulatory regions of *I.trifida* NBS encoding genes.

Table S1: Primers used in qRT-PCR

Primer name	Primer sequence (5′-3′)
IbNBS10-F	ACACAAAACTGCCGTTGAGC
IbNBS10-R	CCCGATGAAGAAAGCCGAGT
IbNBS20-F	GCATGGCCGTAACCAAACAG
IbNBS20-R	AAATCCCCGCGAACACCTAA
IbNBS258-F	CCCGGAGTGGCAATGTAGTT
IbNBS258-R	ACGAGGTCCCTCTGTACCAA
*IbNBS88*F	AAGACCAAAGTGCTACGCGA
IbNBS88-R	ATCCCAATGCTGAGTGCTCC
Actin-F	AGCAGCATGAAGATTAAGGTTGTAGCAC
Actin-R	TGGAAAATTAGAAGCACTTCCTGTGAAC

Table S2: The information of NBS genes in *I. trifida*

NO.	Name	TIR	CC	RPW8	LRR_1	LRR_3	LRR_4	LRR_5	LRR_6	LRR_8	LRR_9	Type	Chromosome	Location	+/-
1	itf00g00070.t1		CC				LRR_4			LRR_8		CNL	Chr00	76410-79837	–
2	itf00g01120.t1						LRR_4			LRR_8		NL	Chr00	1745317-1748522	+
3	itf00g04550.t1		CC									CN	Chr00	7227500-7228756	+
4	itf00g04820.t1											N	Chr00	7815132-7819110	+
5	itf00g07890.t1		CC				LRR_4			LRR_8		CNL	Chr00	13200946-13204810	–
6	itf00g07890.t2		CC				LRR_4			LRR_8		CNL	Chr00	13200946-13204810	–
7	itf00g08100.t1	TIR					LRR_4			LRR_8		TNL	Chr00	13373023-13385815	–
8	itf00g08120.t1	TIR			LRR_1		LRR_4			LRR_8		TNL	Chr00	13399014-13408232	–
9	itf00g08120.t2	TIR			LRR_1		LRR_4			LRR_8		TNL	Chr00	13399014-13408232	–
10	itf00g08140.t1	TIR										TN	Chr00	13420366-13423525	–
11	itf00g08620.t1						LRR_4					NL	Chr00	14374530-14376731	–
12	itf00g08630.t1									LRR_8		NL	Chr00	14379852-14383219	–
13	itf00g08650.t1											N	Chr00	14402023-14403198	–
14	itf00g08680.t1											N	Chr00	14430815-14433388	–
15	itf00g08680.t2											N	Chr00	14430815-14433388	–
16	itf00g08690.t1						LRR_4			LRR_8		NL	Chr00	14455419-14457780	–
17	itf00g10780.t1	TIR			LRR_1	LRR_3	LRR_4			LRR_8	LRR_9	TNL	Chr00	18226280-18230945	+
18	itf00g10780.t2	TIR			LRR_1	LRR_3	LRR_4			LRR_8	LRR_9	TNL	Chr00	18226280-18230945	+
19	itf00g11080.t1	TIR					LRR_4	LRR_5		LRR_8		TNL	Chr00	18747387-18755871	+
20	itf00g11080.t2	TIR					LRR_4	LRR_5		LRR_8		TNL	Chr00	18747387-18755871	+
21	itf00g11080.t3						LRR_4	LRR_5		LRR_8		NL	Chr00	18749351-18755871	+
22	itf00g11230.t1						LRR_4			LRR_8		NL	Chr00	19009961-19019012	–
23	itf00g11230.t2						LRR_4			LRR_8		NL	Chr00	19009961-19019012	–
24	itf00g16800.t1	TIR					LRR_4					TNL	Chr00	28108315-28113161	+
25	itf00g16800.t2	TIR										TN	Chr00	28108315-28111126	+
26	itf00g16810.t1	TIR					LRR_4					TNL	Chr00	28118456-28121765	+
27	itf00g19420.t1	TIR			LRR_1		LRR_4	LRR_5		LRR_8		TNL	Chr00	32368073-32373951	–
28	itf00g19420.t2				LRR_1	LRR_3	LRR_4	LRR_5		LRR_8		NL	Chr00	32368073-32372856	–
29	itf00g20050.t1						LRR_4			LRR_8		NL	Chr00	33384307-33388928	+

(*Continued*)

Table S2: *Continued*

NO.	Name	TIR	CC	RPW8	LRR_1	LRR_3	LRR_4	LRR_5	LRR_6	LRR_8	LRR_9	Type	Chromosome	Location	+/-
30	itf00g20060.t1									LRR_8		NL	Chr00	33411618-33414872	+
31	itf00g20300.t1											N	Chr00	33801513-33802959	+
32	itf00g24790.t1		CC				LRR_4			LRR_8		CNL	Chr00	40883831-40889077	+
33	itf00g24790.t2		CC				LRR_4			LRR_8		CNL	Chr00	40883848-40888988	+
34	itf00g24800.t1		CC							LRR_8		CNL	Chr00	40896454-40904085	—
35	itf00g25710.t1											N	Chr00	42143610-42146386	—
36	itf00g25720.t1											N	Chr00	42159686-42162345	—
37	itf00g29730.t1						LRR_4			LRR_8		NL	Chr00	47658154-47660448	+
38	itf00g29740.t1				LRR_1		LRR_4			LRR_8		NL	Chr00	47661856-47664150	—
39	itf00g30480.t1		CC				LRR_4					CNL	Chr00	48245924-48248707	—
40	itf00g30960.t1		CC				LRR_4			LRR_8		CNL	Chr00	48732158-48737093	—
41	itf00g30960.t2		CC				LRR_4			LRR_8		CNL	Chr00	48732158-48737093	—
42	itf00g30960.t3		CC				LRR_4			LRR_8		CNL	Chr00	48732158-48737093	—
43	itf00g32110.t1		CC									CN	Chr00	49989640-49991660	+
44	itf01g08960.t1											N	Chr01	7833736-7840964	—
45	itf01g10090.t1						LRR_4					NL	Chr01	9417971-9420229	+
46	itf01g10890.t1		CC							LRR_8		CNL	Chr01	10470610-10473201	+
47	itf01g13670.t1											N	Chr01	14174837-14177123	+
48	itf01g18600.t1	TIR										TN	Chr01	19729085-19735535	+
49	itf01g19690.t1		CC		LRR_1		LRR_4			LRR_8		CNL	Chr01	20708617-20711393	—
50	itf01g19840.t1				LRR_1		LRR_4			LRR_8	LRR_9	NL	Chr01	20817063-20819323	—
51	itf01g20080.t1		CC		LRR_1		LRR_4			LRR_8		CNL	Chr01	21033266-21036098	+
52	itf01g20090.t1		CC				LRR_4			LRR_8		CNL	Chr01	21056805-21060138	+
53	itf01g20130.t1		CC		LRR_1		LRR_4			LRR_8		CNL	Chr01	21077357-21080255	+
54	itf01g21600.t1						LRR_4					NL	Chr01	22438999-22441119	+
55	itf01g24580.t1		CC		LRR_1		LRR_4			LRR_8		CNL	Chr01	24688786-24691461	+
56	itf01g33990.t1			RPW8	LRR_1		LRR_4			LRR_8		RNL	Chr01	31037347-31040998	+
57	itf02g01860.t1		CC		LRR_1		LRR_4			LRR_8	LRR_9	CNL	Chr02	2709175-2711909	—

(*Continued*)

Table S2: *Continued*

NO.	Name	TIR	CC	RPW8	LRR_1	LRR_3	LRR_4	LRR_5	LRR_6	LRR_8	LRR_9	Type	Chromosome	Location	+/-
58	itf02g11630.t1		CC		LRR_1		LRR_4		LRR_6	LRR_8		CNL	Chr02	9811851-9817177	+
59	itf02g11640.t1		CC		LRR_1		LRR_4			LRR_8		CNL	Chr02	9821450-9827145	+
60	itf03g12490.t1		CC									CN	Chr03	10830582-10833221	+
61	itf03g20420.t1		CC							LRR_8		CNL	Chr03	16026696-16030043	—
62	itf03g20470.t1									LRR_8		NL	Chr03	16083952-16089225	+
63	itf03g20470.t2									LRR_8		NL	Chr03	16083952-16088994	+
64	itf03g20470.t3									LRR_8		NL	Chr03	16083952-16088933	+
65	itf03g20470.t4									LRR_8		NL	Chr03	16083952-16088349	+
66	itf03g20500.t1		CC							LRR_8		CNL	Chr03	16110206-16116290	+
67	itf03g20560.t1		CC							LRR_8		CNL	Chr03	16161949-16165794	+
68	itf03g20560.t2		CC							LRR_8		CNL	Chr03	16161949-16165794	+
69	itf03g20560.t3		CC							LRR_8		CNL	Chr03	16161949-16165794	+
70	itf03g20730.t1		CC							LRR_8		CNL	Chr03	16363280-16367820	—
71	itf03g20730.t2									LRR_8		NL	Chr03	16363280-16366145	—
72	itf03g20740.t1		CC							LRR_8		CNL	Chr03	16368577-16373245	—
73	itf03g20760.t1		CC									CN	Chr03	16379943-16384641	+
74	itf03g20950.t1				LRR_1		LRR_4					NL	Chr03	16620375-16623566	+
75	itf03g20980.t1		CC		LRR_1							CNL	Chr03	16632110-16635721	+
76	itf03g20990.t1		CC									CN	Chr03	16647035-16649112	—
77	itf03g20990.t2		CC				LRR_4			LRR_8		CNL	Chr03	16644750-16649112	—
78	itf03g21020.t1		CC		LRR_1		LRR_4			LRR_8		CNL	Chr03	16657752-16661645	—
79	itf03g21020.t2		CC		LRR_1		LRR_4			LRR_8		CNL	Chr03	16657752-16661645	—
80	itf03g21700.t1											N	Chr03	17275173-17280199	+
81	itf03g21710.t1											N	Chr03	17283431-17285880	—
82	itf03g21720.t1		CC		LRR_1							CNL	Chr03	17288215-17292400	+
83	itf03g21720.t2		CC		LRR_1							CNL	Chr03	17288215-17292400	+
84	itf03g21730.t1		CC									CN	Chr03	17294688-17298834	+
85	itf03g21750.t1		CC									CN	Chr03	17319727-17322585	+

(*Continued*)

Table S2: *Continued*

NO.	Name	TIR	CC	RPW8	LRR_1	LRR_3	LRR_4	LRR_5	LRR_6	LRR_8	LRR_9	Type	Chromosome	Location	+/-
86	itf03g21770.t1		CC							LRR_8		CNL	Chr03	17328268-17331087	+
87	itf03g22010.t1						LRR_4					NL	Chr03	17491198-17493360	+
88	itf03g22030.t1		CC									CN	Chr03	17506893-17509697	+
89	itf03g22040.t1						LRR_4					NL	Chr03	17512513-17514936	+
90	itf03g22050.t1		CC									CN	Chr03	17517189-17519819	—
91	itf03g22060.t1											N	Chr03	17522234-17524678	—
92	itf03g23010.t1		CC				LRR_4			LRR_8		CNL	Chr03	18504844-18508422	+
93	itf03g27300.t1									LRR_8		NL	Chr03	23584116-23585975	+
94	itf03g27310.t1						LRR_4			LRR_8		NL	Chr03	23586619-23595000	+
95	itf03g27550.t1				LRR_1		LRR_4					NL	Chr03	23923311-23925855	—
96	itf03g27560.t1		CC									CN	Chr03	23928941-23935466	—
97	itf03g28470.t1		CC							LRR_8		CNL	Chr03	25238153-25245441	+
98	itf04g00730.t1		CC									CN	Chr04	339275-342678	+
99	itf04g01350.t1		CC									CN	Chr04	665729-671210	+
100	itf04g01370.t1											N	Chr04	673921-677709	+
101	itf04g01390.t1		CC				LRR_4					CNL	Chr04	686614-694849	+
102	itf04g01390.t2		CC				LRR_4					CNL	Chr04	687114-694849	+
103	itf04g01390.t3		CC									CN	Chr04	686614-690684	+
104	itf04g01390.t4		CC									CN	Chr04	687114-692509	+
105	itf04g01400.t1											N	Chr04	698074-703878	+
106	itf04g04430.t1		CC				LRR_4			LRR_8		CNL	Chr04	2507044-2510068	+
107	itf04g04440.t1		CC				LRR_4			LRR_8		CNL	Chr04	2511131-2513923	+
108	itf04g04450.t1		CC				LRR_4			LRR_8		CNL	Chr04	2517170-2520037	+
109	itf04g05380.t1		CC				LRR_4			LRR_8		CNL	Chr04	3078624-3082094	+
110	itf04g05390.t1		CC							LRR_8		CNL	Chr04	3084628-3089596	—
111	itf04g05400.t1		CC									CN	Chr04	3092265-3095074	+
112	itf04g05410.t1		CC				LRR_4			LRR_8		CNL	Chr04	3098273-3101802	+
113	itf04g05410.t2		CC				LRR_4			LRR_8		CNL	Chr04	3098273-3101802	+

(*Continued*)

Table S2: *Continued*

NO.	Name	TIR	CC	RPW8	LRR_1	LRR_3	LRR_4	LRR_5	LRR_6	LRR_8	LRR_9	Type	Chromosome	Location	+/-
114	itf04g05490.t1		CC		LRR_1		LRR_4			LRR_8		CNL	Chr04	3158910-3163476	–
115	itf04g05490.t2		CC									CN	Chr04	3158910-3163476	–
116	itf04g06680.t1				LRR_1		LRR_4		LRR_6	LRR_8		NL	Chr04	3991440-3992743	–
117	itf04g07270.t1									LRR_8		NL	Chr04	4421826-4425595	+
118	itf04g08290.t1		CC				LRR_4			LRR_8		CNL	Chr04	5230865-5234409	+
119	itf04g08640.t1				LRR_1		LRR_4			LRR_8		NL	Chr04	5539440-5541791	–
120	itf04g08850.t1		CC									CN	Chr04	5751264-5754110	–
121	itf04g09260.t1		CC									CN	Chr04	6134978-6145586	–
122	itf04g09550.t1	TIR										TN	Chr04	6423008-6430473	+
123	itf04g09550.t2											N	Chr04	6424788-6430473	+
124	itf04g09950.t1		CC									CN	Chr04	6830026-6836673	+
125	itf04g09990.t1		CC									CN	Chr04	6861865-6865855	+
126	itf04g09990.t2		CC									CN	Chr04	6861865-6865117	+
127	itf04g10000.t1		CC									CN	Chr04	6868641-6873510	+
128	itf04g10000.t2		CC									CN	Chr04	6868641-6872963	+
129	itf04g10000.t3		CC									CN	Chr04	6868641-6871982	+
130	itf04g10060.t1		CC		LRR_1		LRR_4					CNL	Chr04	6925946-6929505	+
131	itf04g10180.t1		CC				LRR_4					CNL	Chr04	7011500-7014941	–
132	itf04g10190.t1				LRR_1		LRR_4			LRR_8		NL	Chr04	7020072-7023076	–
133	itf04g10200.t1		CC									CN	Chr04	7028684-7031057	–
134	itf04g13450.t1		CC		LRR_1		LRR_4					CNL	Chr04	10869151-10871718	+
135	itf04g13460.t1		CC				LRR_4			LRR_8		CNL	Chr04	10876980-10879604	+
136	itf04g13470.t1		CC				LRR_4					CNL	Chr04	10900093-10902675	+
137	itf04g34220.t1		CC				LRR_4					CNL	Chr04	32237671-32240253	+
138	itf04g34230.t1		CC				LRR_4			LRR_8		CNL	Chr04	32241344-32243965	–
139	itf04g34280.t1		CC				LRR_4			LRR_8		CNL	Chr04	32278733-32282551	+
140	itf05g06500.t1		CC				LRR_4			LRR_8		CNL	Chr05	6287326-6294369	–
141	itf05g06500.t2		CC				LRR_4			LRR_8		CNL	Chr05	6287326-6294369	–

(*Continued*)

Table S2: *Continued*

NO.	Name	TIR	CC	RPW8	LRR_1	LRR_3	LRR_4	LRR_5	LRR_6	LRR_8	LRR_9	Type	Chromosome	Location	+/-
142	itf05g06510.t1		CC		LRR_1		LRR_4			LRR_8		CNL	Chr05	6338581-6345194	+
143	itf05g06510.t2		CC		LRR_1		LRR_4			LRR_8		CNL	Chr05	6338581-6345194	+
144	itf05g08990.t1				LRR_1							NL	Chr05	10177697-10178896	+
145	itf05g11580.t1		CC									CN	Chr05	14119180-14120613	–
146	itf06g03570.t1						LRR_4			LRR_8		NL	Chr06	5323186-5325786	+
147	itf06g05520.t1						LRR_4			LRR_8		NL	Chr06	7709752-7712148	–
148	itf06g05600.t1				LRR_1		LRR_4			LRR_8		NL	Chr06	7763659-7766118	–
149	itf06g06580.t1						LRR_4			LRR_8		NL	Chr06	9004001-9010736	+
150	itf06g09970.t1											N	Chr06	12442170-12445100	–
151	itf06g10010.t1						LRR_4			LRR_8		NL	Chr06	12485454-12487994	–
152	itf06g10030.t1						LRR_4	LRR_5	LRR_6	LRR_8		NL	Chr06	12580273-12585111	–
153	itf06g10030.t2						LRR_4	LRR_5	LRR_6	LRR_8		NL	Chr06	12580273-12585111	–
154	itf06g10050.t1				LRR_1		LRR_4			LRR_8		NL	Chr06	12602873-12605440	–
155	itf06g10110.t1						LRR_4			LRR_8		NL	Chr06	12660783-12665398	–
156	itf06g10110.t2						LRR_4			LRR_8		NL	Chr06	12660783-12665398	–
157	itf06g10580.t1						LRR_4			LRR_8		NL	Chr06	13283085-13285634	+
158	itf06g12150.t1		CC		LRR_1		LRR_4			LRR_8		CNL	Chr06	15086533-15089472	+
159	itf06g12180.t1		CC		LRR_1		LRR_4			LRR_8		CNL	Chr06	15122832-15129525	+
160	itf06g12180.t2		CC		LRR_1		LRR_4			LRR_8		CNL	Chr06	15122832-15129525	+
161	itf06g12180.t3		CC		LRR_1		LRR_4			LRR_8		CNL	Chr06	15122832-15129525	+
162	itf06g12180.t4		CC		LRR_1		LRR_4			LRR_8		CNL	Chr06	15122832-15129525	+
163	itf06g12180.t5		CC		LRR_1		LRR_4			LRR_8		CNL	Chr06	15122832-15127982	+
164	itf06g12180.t6		CC		LRR_1		LRR_4			LRR_8		CNL	Chr06	15122832-15127982	+
165	itf06g12180.t7		CC		LRR_1		LRR_4			LRR_8		CNL	Chr06	15122830-15127982	+
166	itf06g12180.t8		CC		LRR_1		LRR_4			LRR_8		CNL	Chr06	15122832-15127982	+
167	itf06g12210.t1		CC		LRR_1		LRR_4			LRR_8		CNL	Chr06	15174064-15181425	+
168	itf06g12210.t2		CC		LRR_1		LRR_4			LRR_8		CNL	Chr06	15177154-15181425	+
169	itf06g12250.t1		CC		LRR_1		LRR_4			LRR_8		CNL	Chr06	15214098-15218969	+

(*Continued*)

Table S2: *Continued*

NO.	Name	TIR	CC	RPW8	LRR_1	LRR_3	LRR_4	LRR_5	LRR_6	LRR_8	LRR_9	Type	Chromosome	Location	+/-
170	itf06g12250.t2		CC		LRR_1		LRR_4			LRR_8		CNL	Chr06	15214220-15218969	+
171	itf06g12270.t1		CC		LRR_1		LRR_4			LRR_8		CNL	Chr06	15247153-15250071	+
172	itf06g12280.t1						LRR_4			LRR_8		NL	Chr06	15266818-15269670	+
173	itf06g12290.t1		CC				LRR_4			LRR_8		CNL	Chr06	15303259-15307865	+
174	itf06g12350.t1				LRR_1		LRR_4		LRR_6	LRR_8		NL	Chr06	15342989-15345847	+
175	itf06g13050.t1									LRR_8		NL	Chr06	16152012-16154909	+
176	itf06g13060.t1						LRR_4					NL	Chr06	16172555-16175713	+
177	itf06g13060.t2						LRR_4					NL	Chr06	16172555-16175713	+
178	itf06g13060.t3						LRR_4					NL	Chr06	16172555-16175713	+
179	itf06g13060.t4						LRR_4					NL	Chr06	16172555-16175713	+
180	itf06g13070.t1		CC				LRR_4					CNL	Chr06	16218626-16221846	+
181	itf06g13070.t2		CC				LRR_4					CNL	Chr06	16218626-16221846	+
182	itf06g13070.t3		CC				LRR_4					CNL	Chr06	16218626-16221846	+
183	itf06g13090.t1		CC				LRR_4					CNL	Chr06	16258932-16262342	+
184	itf06g13100.t1		CC				LRR_4					CNL	Chr06	16263645-16266841	+
185	itf06g13100.t2		CC				LRR_4					CNL	Chr06	16263645-16266841	+
186	itf06g13100.t3		CC				LRR_4					CNL	Chr06	16263645-16266841	+
187	itf06g13610.t1				LRR_1		LRR_4			LRR_8		NL	Chr06	16750994-16756825	+
188	itf06g15880.t1		CC									CN	Chr06	19005659-19010970	+
189	itf06g20590.t1		CC				LRR_4			LRR_8		CNL	Chr06	22334955-22338606	+
190	itf06g20590.t3		CC				LRR_4			LRR_8		CNL	Chr06	22335043-22337775	+
191	itf07g02310.t1	TIR					LRR_4		LRR_6	LRR_8		TNL	Chr07	1366589-1384114	—
192	itf07g02310.t2	TIR					LRR_4		LRR_6	LRR_8		TNL	Chr07	1367097-1384114	—
193	itf07g02310.t3	TIR					LRR_4		LRR_6	LRR_8		TNL	Chr07	1367097-1384114	—
194	itf07g02310.t4	TIR					LRR_4		LRR_6	LRR_8		TNL	Chr07	1371753-1384114	—
195	itf07g02310.t5	TIR					LRR_4		LRR_6	LRR_8		TNL	Chr07	1367097-1384114	—
196	itf07g02370.t1	TIR					LRR_4			LRR_8		TNL	Chr07	1486353-1496878	—
197	itf07g02370.t2	TIR					LRR_4			LRR_8		TNL	Chr07	1486353-1496878	—

(*Continued*)

Table S2: *Continued*

NO.	Name	TIR	CC	RPW8	LRR_1	LRR_3	LRR_4	LRR_5	LRR_6	LRR_8	LRR_9	Type	Chromosome	Location	+/-
198	itf07g02380.t1	TIR					LRR_4			LRR_8		TNL	Chr07	1499531-1521536	—
199	itf07g02380.t2	TIR					LRR_4			LRR_8		TNL	Chr07	1499531-1521536	—
200	itf07g02380.t3	TIR					LRR_4			LRR_8		TNL	Chr07	1499531-1521536	—
201	itf07g02520.t1	TIR					LRR_4			LRR_8		TNL	Chr07	1581264-1603111	—
202	itf07g02520.t2	TIR					LRR_4			LRR_8		TNL	Chr07	1581264-1603111	—
203	itf07g07830.t1		CC									CN	Chr07	5647364-5653067	—
204	itf07g07850.t1		CC							LRR_8		CNL	Chr07	5667761-5671983	—
205	itf07g07860.t1		CC									CN	Chr07	5686084-5693337	—
206	itf07g07860.t2		CC									CN	Chr07	5686087-5693333	—
207	itf07g07880.t1		CC				LRR_4			LRR_8		CNL	Chr07	5715639-5722125	—
208	itf07g07900.t1		CC				LRR_4					CNL	Chr07	5732595-5735515	—
209	itf07g07910.t1		CC		LRR_1					LRR_8		CNL	Chr07	5738794-5746695	—
210	itf07g07910.t2				LRR_1		LRR_4			LRR_8		NL	Chr07	5738794-5742045	—
211	itf07g09670.t1											N	Chr07	7688254-7688967	—
212	itf07g11720.t1											N	Chr07	10334036-10346730	—
213	itf07g14280.t1											N	Chr07	13698407-13706832	—
214	itf07g16420.t1						LRR_4			LRR_8		NL	Chr07	16582347-16603085	—
215	itf07g16420.t2						LRR_4			LRR_8		NL	Chr07	16582347-16603085	—
216	itf07g16420.t4						LRR_4			LRR_8		NL	Chr07	16582347-16603085	—
217	itf07g16420.t5						LRR_4			LRR_8		NL	Chr07	16582347-16603085	—
218	itf07g16420.t6						LRR_4			LRR_8		NL	Chr07	16590833-16603085	—
219	itf07g16420.t7						LRR_4			LRR_8		NL	Chr07	16586915-16603085	—
220	itf07g16420.t8						LRR_4			LRR_8		NL	Chr07	16591566-16603085	—
221	itf07g16830.t1											N	Chr07	16994491-16997674	—
222	itf07g20970.t1	TIR					LRR_4	LRR_5		LRR_8		TNL	Chr07	21466020-21470582	—
223	itf07g20970.t2	TIR					LRR_4			LRR_8		TNL	Chr07	21466020-21470582	—
224	itf07g20970.t3	TIR					LRR_4	LRR_5		LRR_8		TNL	Chr07	21467319-21470582	—
225	itf07g22370.t1		CC									CN	Chr07	22647964-22649640	+

(*Continued*)

Table S2: *Continued*

NO.	Name	TIR	CC	RPW8	LRR_1	LRR_3	LRR_4	LRR_5	LRR_6	LRR_8	LRR_9	Type	Chromosome	Location	+/-
226	itf07g22420.t1		CC									CN	Chr07	22674993-22677930	+
227	itf07g22430.t1		CC									CN	Chr07	22680554-22681864	+
228	itf07g22440.t1		CC									CN	Chr07	22688058-22690637	+
229	itf07g22460.t1											N	Chr07	22696215-22697699	–
230	itf07g22470.t1		CC									CN	Chr07	22701519-22702862	+
231	itf08g09480.t2	TIR					LRR_4			LRR_8		TNL	Chr08	7494270-7503109	+
232	itf08g09480.t3	TIR					LRR_4	LRR_5		LRR_8		TNL	Chr08	7494270-7503109	+
233	itf08g09480.t4						LRR_4	LRR_5		LRR_8		NL	Chr08	7497536-7503109	+
234	itf08g09750.t1	TIR					LRR_4	LRR_5		LRR_8		TNL	Chr08	7810412-7815118	–
235	itf08g12730.t1	TIR			LRR_1		LRR_4	LRR_5		LRR_8	LRR_9	TNL	Chr08	11684827-11693230	–
236	itf08g12730.t2				LRR_1		LRR_4	LRR_5		LRR_8	LRR_9	NL	Chr08	11684827-11693223	–
237	itf08g12730.t3	TIR			LRR_1		LRR_4	LRR_5		LRR_8	LRR_9	TNL	Chr08	11685928-11693225	–
238	itf08g12730.t4				LRR_1		LRR_4	LRR_5		LRR_8	LRR_9	NL	Chr08	11684827-11689925	–
239	itf08g12730.t5				LRR_1		LRR_4	LRR_5		LRR_8	LRR_9	NL	Chr08	11685928-11689925	–
240	itf08g14610.t1	TIR					LRR_4		LRR_6	LRR_8		TNL	Chr08	14178312-14186529	+
241	itf08g14640.t1	TIR					LRR_4		LRR_6	LRR_8		TNL	Chr08	14204096-14230582	+
242	itf08g17370.t1		CC									CN	Chr08	17347700-17350120	+
243	itf08g17400.t1		CC									CN	Chr08	17415705-17419852	+
244	itf08g17410.t1						LRR_4					NL	Chr08	17426717-17428922	+
245	itf09g07950.t1		CC				LRR_4			LRR_8		CNL	Chr09	4264741-4270265	+
246	itf09g16380.t1	TIR					LRR_4	LRR_5		LRR_8		TNL	Chr09	10986437-10992235	–
247	itf09g16380.t2	TIR					LRR_4	LRR_5		LRR_8		TNL	Chr09	10986437-10992235	–
248	itf09g17780.t1											N	Chr09	12932959-12939664	+
249	itf09g21670.t1				LRR_1		LRR_4			LRR_8		NL	Chr09	18369380-18371671	+
250	itf09g24730.t1		CC		LRR_1		LRR_4			LRR_8		CNL	Chr09	21190508-21193588	–
251	itf09g24740.t1				LRR_1		LRR_4			LRR_8		NL	Chr09	21221514-21223937	–
252	itf09g24750.t1		CC		LRR_1		LRR_4		LRR_6	LRR_8		CNL	Chr09	21232671-21238045	–
253	itf09g24770.t1		CC				LRR_4			LRR_8		CNL	Chr09	21257989-21263491	–

(*Continued*)

Table S2: *Continued*

NO.	Name	TIR	CC	RPW8	LRR_1	LRR_3	LRR_4	LRR_5	LRR_6	LRR_8	LRR_9	Type	Chromosome	Location	+/-
254	itf09g24780.t1		CC				LRR_4			LRR_8		CNL	Chr09	21298046-21305384	–
255	itf09g24780.t2		CC				LRR_4			LRR_8		CNL	Chr09	21298046-21305029	–
256	itf09g24790.t1		CC		LRR_1		LRR_4			LRR_8		CNL	Chr09	21321045-21324094	–
257	itf09g24810.t1		CC									CN	Chr09	21352913-21354264	–
258	itf09g24820.t1		CC				LRR_4			LRR_8		CNL	Chr09	21358148-21362805	–
259	itf09g24850.t1		CC				LRR_4			LRR_8		CNL	Chr09	21381344-21384034	–
260	itf09g24950.t1		CC				LRR_4			LRR_8		CNL	Chr09	21477124-21479982	+
261	itf09g24960.t1		CC		LRR_1		LRR_4		LRR_6	LRR_8		CNL	Chr09	21485524-21488301	+
262	itf09g24980.t1		CC				LRR_4			LRR_8		CNL	Chr09	21495805-21498585	+
263	itf09g24990.t1		CC				LRR_4			LRR_8		CNL	Chr09	21535465-21538276	+
264	itf09g25000.t1		CC				LRR_4			LRR_8		CNL	Chr09	21561985-21571327	+
265	itf09g25000.t2		CC				LRR_4			LRR_8		CNL	Chr09	21567910-21571327	+
266	itf09g25590.t1		CC				LRR_4			LRR_8		CNL	Chr09	22238833-22241610	–
267	itf09g25610.t1		CC				LRR_4			LRR_8		CNL	Chr09	22251586-22254375	–
268	itf10g05570.t1		CC				LRR_4			LRR_8		CNL	Chr10	5484003-5486306	–
269	itf10g05780.t1	TIR			LRR_1	LRR_3	LRR_4		LRR_6	LRR_8	LRR_9	TNL	Chr10	5756753-5767090	+
270	itf10g07890.t1											N	Chr10	8662674-8670760	+
271	itf10g12820.t1						LRR_4					NL	Chr10	15421449-15423632	+
272	itf11g05970.t1		CC				LRR_4			LRR_8		CNL	Chr11	3107596-3111021	+
273	itf11g13930.t1				LRR_1		LRR_4			LRR_8		NL	Chr11	9825446-9828047	+
274	itf11g13960.t1											N	Chr11	9842326-9845030	+
275	itf11g18990.t1		CC		LRR_1		LRR_4			LRR_8		CNL	Chr11	16334983-16338241	+
276	itf11g20040.t1		CC		LRR_1		LRR_4		LRR_6	LRR_8		CNL	Chr11	17367395-17371968	–
277	itf11g20040.t2		CC		LRR_1		LRR_4		LRR_6	LRR_8		CNL	Chr11	17367395-17371968	–
278	itf11g20040.t3		CC		LRR_1		LRR_4		LRR_6	LRR_8		CNL	Chr11	17367395-17371968	–
279	itf11g20050.t1				LRR_1		LRR_4			LRR_8		NL	Chr11	17384431-17386623	–
280	itf11g20070.t1											N	Chr11	17402253-17403498	–
281	itf11g20070.t2											N	Chr11	17401590-17403498	–

(*Continued*)

Table S2: *Continued*

NO.	Name	TIR	CC	RPW8	LRR_1	LRR_3	LRR_4	LRR_5	LRR_6	LRR_8	LRR_9	Type	Chromosome	Location	+/-
282	itf11g20080.t1											N	Chr11	17405979-17408574	–
283	itf11g20280.t1											N	Chr11	17629866-17632275	–
284	itf11g20290.t1											N	Chr11	17637318-17639800	–
285	itf11g20300.t1											N	Chr11	17644440-17646216	–
286	itf11g20310.t1				LRR_1		LRR_4			LRR_8		NL	Chr11	17653714-17656116	–
287	itf11g20320.t1											N	Chr11	17660866-17663083	–
288	itf11g20430.t1		CC		LRR_1							CNL	Chr11	17759774-17765535	+
289	itf11g20430.t2		CC		LRR_1							CNL	Chr11	17759774-17765535	+
290	itf11g20430.t3		CC		LRR_1							CNL	Chr11	17761318-17765535	+
291	itf11g20430.t4		CC		LRR_1							CNL	Chr11	17759664-17765535	+
292	itf11g20440.t1		CC		LRR_1		LRR_4			LRR_8		CNL	Chr11	17768094-17771927	+
293	itf11g20440.t2		CC		LRR_1		LRR_4			LRR_8		CNL	Chr11	17768094-17771927	+
294	itf11g20450.t1				LRR_1		LRR_4					NL	Chr11	17774232-17777200	+
295	itf11g20470.t1											N	Chr11	17787857-17793601	+
296	itf11g20480.t1											N	Chr11	17797000-17799678	+
297	itf11g20490.t1				LRR_1		LRR_4			LRR_8		NL	Chr11	17807103-17811054	+
298	itf11g20510.t1		CC		LRR_1		LRR_4					CNL	Chr11	17840936-17844711	+
299	itf11g20570.t1		CC									CN	Chr11	17900720-17904111	+
300	itf11g20580.t1						LRR_4					NL	Chr11	17908979-17911296	+
301	itf11g20590.t1											N	Chr11	17913976-17917189	+
302	itf11g20610.t1				LRR_1		LRR_4			LRR_8		NL	Chr11	17936326-17938743	+
303	itf11g20650.t1											N	Chr11	17999088-18001524	–
304	itf11g20660.t1						LRR_4					NL	Chr11	18003442-18006655	–
305	itf11g20670.t1		CC				LRR_4					CNL	Chr11	18007319-18010954	–
306	itf11g21040.t1				LRR_1		LRR_4			LRR_8		NL	Chr11	18311634-18315562	+
307	itf11g21790.t1											N	Chr11	19050936-19052813	–
308	itf11g21840.t1				LRR_1		LRR_4			LRR_8		NL	Chr11	19133969-19136335	+
309	itf12g05370.t1			RPW8	LRR_1					LRR_8		RNL	Chr12	3300900-3305525	–

(*Continued*)

Table S2: *Continued*

NO.	Name	TIR	CC	RPW8	LRR_1	LRR_3	LRR_4	LRR_5	LRR_6	LRR_8	LRR_9	Type	Chromosome	Location	+/-
310	itf12g13120.t1						LRR_4					NL	Chr12	10400406-10405846	–
311	itf12g13120.t2						LRR_4					NL	Chr12	10400406-10404056	–
312	itf12g13120.t3						LRR_4					NL	Chr12	10401357-10404056	–
313	itf12g21200.t1	TIR										TN	Chr12	20232441-20234527	+
314	itf12g21480.t1	TIR										TN	Chr12	20457780-20462388	–
315	itf13g00800.t1						LRR_4			LRR_8		NL	Chr13	580780-584264	+
316	itf13g00810.t1											N	Chr13	592669-596766	+
317	itf13g00840.t1											N	Chr13	610427-613148	+
318	itf13g00960.t1		CC							LRR_8		CNL	Chr13	724575-728003	–
319	itf13g01010.t1		CC									CN	Chr13	778959-783675	+
320	itf13g01030.t1		CC				LRR_4					CNL	Chr13	796780-801798	+
321	itf13g01450.t1		CC									CN	Chr13	1099547-1103584	–
322	itf13g01470.t1		CC				LRR_4			LRR_8		CNL	Chr13	1110145-1113494	–
323	itf13g01480.t1		CC									CN	Chr13	1126330-1130980	–
324	itf13g01590.t1						LRR_4			LRR_8		NL	Chr13	1202219-1205068	–
325	itf13g01670.t1		CC									CN	Chr13	1252725-1255872	+
326	itf13g01680.t1		CC							LRR_8		CNL	Chr13	1256570-1262130	+
327	itf13g01680.t2		CC							LRR_8		CNL	Chr13	1256570-1262130	+
328	itf13g01700.t1		CC									CN	Chr13	1268062-1271847	–
329	itf13g01710.t1		CC									CN	Chr13	1283899-1287333	+
330	itf13g01710.t2		CC		LRR_1		LRR_4			LRR_8		CNL	Chr13	1283899-1287342	+
331	itf13g01720.t1		CC				LRR_4			LRR_8		CNL	Chr13	1292802-1295846	+
332	itf13g01730.t1		CC				LRR_4					CNL	Chr13	1296833-1300478	–
333	itf13g01750.t1		CC				LRR_4					CNL	Chr13	1311114-1314889	–
334	itf13g01750.t2		CC									CN	Chr13	1311114-1314889	–
335	itf13g02070.t1		CC							LRR_8		CNL	Chr13	1612877-1615958	–
336	itf13g02090.t1		CC				LRR_4					CNL	Chr13	1628922-1631910	–
337	itf13g02090.t2		CC				LRR_4					CNL	Chr13	1628677-1631910	–

(*Continued*)

Table S2: *Continued*

NO.	Name	TIR	CC	RPW8	LRR_1	LRR_3	LRR_4	LRR_5	LRR_6	LRR_8	LRR_9	Type	Chromosome	Location	+/-
338	itf13g02100.t1		CC				LRR_4			LRR_8		CNL	Chr13	1634644-1638544	–
339	itf13g02100.t2		CC				LRR_4			LRR_8		CNL	Chr13	1634644-1638544	–
340	itf13g02100.t3		CC				LRR_4			LRR_8		CNL	Chr13	1634644-1638544	–
341	itf13g02100.t4		CC				LRR_4			LRR_8		CNL	Chr13	1634644-1638544	–
342	itf13g02410.t1		CC				LRR_4					CNL	Chr13	1943162-1947555	–
343	itf13g02540.t1		CC		LRR_1		LRR_4					CNL	Chr13	2061465-2064071	–
344	itf13g02550.t1		CC									CN	Chr13	2069309-2073890	+
345	itf13g03690.t1		CC		LRR_1		LRR_4			LRR_8		CNL	Chr13	3278529-3283664	–
346	itf13g03690.t2		CC		LRR_1		LRR_4			LRR_8		CNL	Chr13	3280317-3283664	–
347	itf13g03710.t1		CC		LRR_1		LRR_4			LRR_8		CNL	Chr13	3298174-3301212	–
348	itf13g03750.t1		CC				LRR_4			LRR_8		CNL	Chr13	3339186-3343969	–
349	itf13g03950.t1		CC		LRR_1		LRR_4			LRR_8		CNL	Chr13	3588614-3594336	+
350	itf13g03970.t1						LRR_4		LRR_6	LRR_8		NL	Chr13	3601948-3604743	+
351	itf13g03980.t1		CC							LRR_8		CNL	Chr13	3609050-3612965	+
352	itf13g04870.t1		CC									CN	Chr13	4584496-4587653	+
353	itf13g05860.t1						LRR_4					NL	Chr13	6446864-6449128	–
354	itf13g12870.t1		CC				LRR_4			LRR_8		CNL	Chr13	15344570-15347596	+
355	itf13g14120.t1				LRR_1		LRR_4			LRR_8		NL	Chr13	16395928-16401499	–
356	itf13g14120.t2				LRR_1		LRR_4			LRR_8		NL	Chr13	16395928-16401499	–
357	itf13g14140.t1		CC				LRR_4					CNL	Chr13	16436471-16439325	–
358	itf13g21940.t1									LRR_8		NL	Chr13	22233777-22237116	+
359	itf13g21940.t2									LRR_8		NL	Chr13	22233777-22237116	+
360	itf13g21940.t3									LRR_8		NL	Chr13	22233777-22237116	+
361	itf13g21940.t4									LRR_8		NL	Chr13	22233777-22237116	+
362	itf13g21940.t5									LRR_8		NL	Chr13	22233777-22237116	+
363	itf13g21940.t6									LRR_8		NL	Chr13	22233777-22237116	+
364	itf13g21950.t1									LRR_8		NL	Chr13	22245970-22248171	+
365	itf13g21960.t1						LRR_4					NL	Chr13	22248721-22250907	+

(*Continued*)

Table S2: *Continued*

NO.	Name	TIR	CC	RPW8	LRR_1	LRR_3	LRR_4	LRR_5	LRR_6	LRR_8	LRR_9	Type	Chromosome	Location	+/-
366	itf13g21990.t1									LRR_8		NL	Chr13	22261565-22266979	+
367	itf13g21990.t2									LRR_8		NL	Chr13	22264230-22266979	+
368	itf14g00240.t1		CC				LRR_4			LRR_8		CNL	Chr14	187407-190076	+
369	itf14g00250.t1				LRR_1		LRR_4					NL	Chr14	193413-196674	+
370	itf14g00270.t1		CC				LRR_4			LRR_8		CNL	Chr14	201705-205222	—
371	itf14g01400.t1		CC							LRR_8		CNL	Chr14	1120653-1130193	—
372	itf14g01400.t2									LRR_8		NL	Chr14	1120672-1125833	—
373	itf14g01400.t3		CC							LRR_8		CNL	Chr14	1122870-1130072	—
374	itf14g01610.t1		CC				LRR_4			LRR_8		CNL	Chr14	1339570-1349119	—
375	itf14g01620.t1		CC				LRR_4					CNL	Chr14	1353889-1362206	+
376	itf14g01620.t2		CC				LRR_4					CNL	Chr14	1353889-1362206	+
377	itf14g01620.t3		CC				LRR_4					CNL	Chr14	1353899-1362170	+
378	itf14g01630.t1						LRR_4			LRR_8		NL	Chr14	1363757-1369368	+
379	itf14g01640.t1		CC							LRR_8		CNL	Chr14	1369966-1375912	+
380	itf14g01640.t2		CC							LRR_8		CNL	Chr14	1369966-1375062	+
381	itf14g01640.t3						LRR_4			LRR_8		NL	Chr14	1372547-1375853	+
382	itf14g02030.t1						LRR_4			LRR_8		NL	Chr14	1694705-1709185	—
383	itf14g02040.t1									LRR_8		NL	Chr14	1718883-1731583	—
384	itf14g02050.t1									LRR_8		NL	Chr14	1734825-1741132	—
385	itf14g02060.t1				LRR_1					LRR_8		NL	Chr14	1741558-1743616	+
386	itf14g02070.t1									LRR_8		NL	Chr14	1745636-1747981	—
387	itf14g02080.t1		CC							LRR_8		CNL	Chr14	1749907-1756201	—
388	itf14g02090.t1		CC				LRR_4			LRR_8		CNL	Chr14	1766557-1771514	+
389	itf14g02100.t1		CC							LRR_8		CNL	Chr14	1774526-1782721	+
390	itf14g02110.t1		CC									CN	Chr14	1783323-1788637	—
391	itf14g02120.t1		CC				LRR_4			LRR_8		CNL	Chr14	1789398-1792924	+
392	itf14g02290.t1		CC									CN	Chr14	1985375-1988171	—
393	itf14g03840.t1						LRR_4					NL	Chr14	3207203-3210159	+

(*Continued*)

Table S2: *Continued*

NO.	Name	TIR	CC	RPW8	LRR_1	LRR_3	LRR_4	LRR_5	LRR_6	LRR_8	LRR_9	Type	Chromosome	Location	+/-
394	itf14g03860.t1											N	Chr14	3214280-3217105	+
395	itf14g03870.t1						LRR_4					NL	Chr14	3224231-3227194	+
396	itf14g04500.t1		CC		LRR_1		LRR_4			LRR_8		CNL	Chr14	3676776-3681242	+
397	itf14g04500.t2		CC		LRR_1		LRR_4			LRR_8		CNL	Chr14	3677772-3681253	+
398	itf14g04520.t1		CC		LRR_1		LRR_4			LRR_8		CNL	Chr14	3684021-3686741	+
399	itf14g04720.t1		CC									CN	Chr14	3810499-3812528	–
400	itf14g04850.t1		CC		LRR_1		LRR_4			LRR_8		CNL	Chr14	3884020-3886651	+
401	itf14g06890.t1		CC									CN	Chr14	5466865-5469555	+
402	itf14g08270.t1						LRR_4			LRR_8		NL	Chr14	6787761-6793395	+
403	itf15g00150.t1		CC									CN	Chr15	72644-74062	+
404	itf15g04690.t1						LRR_4			LRR_8		NL	Chr15	2786444-2790584	+
405	itf15g10700.t1		CC							LRR_8		CNL	Chr15	7552325-7562997	–
406	itf15g10700.t2		CC							LRR_8		CNL	Chr15	7551181-7562997	–
407	itf15g10770.t1		CC							LRR_8		CNL	Chr15	7593558-7604323	–
408	itf15g10780.t1		CC		LRR_1					LRR_8		CNL	Chr15	7608136-7637238	–
409	itf15g10800.t1		CC							LRR_8		CNL	Chr15	7642976-7656864	–
410	itf15g10800.t2		CC							LRR_8		CNL	Chr15	7643770-7656864	–
411	itf15g10820.t1		CC				LRR_4			LRR_8		CNL	Chr15	7664889-7674252	–
412	itf15g10850.t1		CC		LRR_1					LRR_8		CNL	Chr15	7697571-7718363	–
413	itf15g10880.t1		CC		LRR_1					LRR_8		CNL	Chr15	7726123-7748338	–
414	itf15g17060.t2	TIR										TN	Chr15	15657017-15660822	–
415	itf15g18430.t1						LRR_4			LRR_8		NL	Chr15	17392457-17396117	–
416	itf15g18440.t1						LRR_4					NL	Chr15	17414371-17419660	–
417	itf15g18510.t1		CC		LRR_1		LRR_4			LRR_8		CNL	Chr15	17505196-17507580	+
418	itf15g18540.t1											N	Chr15	17522399-17523475	+
419	itf15g18560.t1				LRR_1		LRR_4					NL	Chr15	17552607-17559568	+
420	itf15g19820.t1				LRR_1					LRR_8		NL	Chr15	18835196-18844594	+
421	itf15g19820.t2				LRR_1					LRR_8		NL	Chr15	18835196-18842199	+

(*Continued*)

Table S2: *Continued*

NO.	Name	TIR	CC	RPW8	LRR_1	LRR_3	LRR_4	LRR_5	LRR_6	LRR_8	LRR_9	Type	Chromosome	Location	+/-
422	itf15g19840.t1		CC							LRR_8		CNL	Chr15	18889155-18900925	+
423	itf15g19850.t1									LRR_8	LRR_9	NL	Chr15	18909215-18915712	+
424	itf15g21480.t3	TIR										TN	Chr15	20585122-20589049	+
425	itf15g21910.t1				LRR_1					LRR_8		NL	Chr15	21033420-21036588	–
426	itf15g21980.t1		CC									CN	Chr15	21069532-21072502	+
427	itf15g22000.t1		CC									CN	Chr15	21077498-21080479	+
428	itf15g22020.t1									LRR_8		NL	Chr15	21092153-21095019	+
429	itf15g22050.t1											N	Chr15	21122535-21126097	+
430	itf15g22060.t1											N	Chr15	21130745-21135351	+
431	itf15g22190.t1		CC							LRR_8		CNL	Chr15	21221989-21225200	–
432	itf15g22210.t1		CC									CN	Chr15	21230343-21240411	–
433	itf15g22210.t2											N	Chr15	21230343-21233423	–
434	itf15g22420.t1											N	Chr15	21407569-21409711	–
435	itf15g22460.t1		CC		LRR_1					LRR_8		CNL	Chr15	21415802-21419228	–
436	itf15g22460.t3		CC		LRR_1					LRR_8		CNL	Chr15	21415873-21419228	–
437	itf15g22470.t1									LRR_8		NL	Chr15	21419996-21422263	+
438	itf15g22490.t1		CC									CN	Chr15	21428510-21432270	–
439	itf15g22510.t1		CC									CN	Chr15	21444954-21447450	+
440	itf15g22520.t1									LRR_8		NL	Chr15	21449093-21451872	–
441	itf15g22540.t1											N	Chr15	21463824-21466481	+
442	itf15g22550.t1											N	Chr15	21477892-21480579	+

Table S3: The number of aa and exons of *I.trifida* NBS genes

Gene	No. of aa	No. of Exons	Type
itf11g20470.t1	541	2	N
itf08g09750.t1	1178	5	TNL
itf11g20310.t1	422	3	NL
itf03g21710.t1	631	3	N
itf15g22460.t3	907	2	CNL
itf00g24790.t2	875	3	CNL
itf04g01390.t4	602	3	CN
itf00g20050.t1	706	5	NL
itf04g06680.t1	413	1	NL
itf14g02080.t1	1085	2	CNL
itf06g10050.t1	856	1	NL
itf08g17400.t1	557	4	CN
itf03g21020.t2	1056	4	CNL
itf04g13460.t1	875	1	CNL
itf03g20420.t1	543	3	CNL
itf13g02100.t4	889	3	CNL
itf07g16420.t5	2125	24	NL
itf14g01610.t1	1704	5	CNL
itf00g04550.t1	419	1	CN
itf14g02050.t1	1243	2	NL
itf00g08620.t1	734	1	NL
itf09g16380.t1	1032	5	TNL
itf09g24950.t1	920	1	CNL
itf07g16420.t1	2032	26	NL
itf05g08990.t1	400	1	NL
itf11g20450.t1	659	2	NL
itf04g01390.t3	576	2	CN
itf00g19420.t2	714	4	NL
itf08g17410.t1	344	6	NL
itf07g22470.t1	448	1	CN
itf04g00730.t1	496	2	CN
itf09g24810.t1	424	2	CN
itf06g12180.t5	987	3	CNL
itf15g10850.t1	2222	11	CNL
itf14g04850.t1	667	3	CNL
itf00g08140.t1	441	2	TN
itf05g06510.t1	941	4	CNL
itf00g11080.t1	1224	7	TNL
itf00g30480.t1	928	1	CNL
itf13g01590.t1	835	2	NL
itf11g20070.t2	421	2	N

Table S3: *Continued*

Gene	No. of aa	No. of Exons	Type
itf15g22540.t1	619	3	N
itf06g10030.t1	985	2	NL
itf13g05860.t1	549	2	NL
itf14g08270.t1	785	4	NL
itf10g05570.t1	768	1	CNL
itf01g13670.t1	450	3	N
itf03g20980.t1	806	5	CNL
itf06g12350.t1	953	1	NL
itf03g27560.t1	557	2	CN
itf11g20430.t4	938	4	CNL
itf04g13470.t1	861	1	CNL
itf04g09950.t1	730	4	CN
itf12g21200.t1	547	2	TN
itf00g08680.t1	395	3	N
itf13g21990.t1	726	3	NL
itf04g05410.t1	918	3	CNL
itf07g16420.t4	2042	25	NL
itf03g20990.t1	666	2	CN
itf08g12730.t2	1147	5	NL
itf06g15880.t1	832	4	CN
itf04g04450.t1	956	1	CNL
itf07g11720.t1	545	5	N
itf12g13120.t3	729	1	NL
itf07g07880.t1	878	3	CNL
itf06g12180.t3	968	4	CNL
itf06g12250.t2	886	4	CNL
itf03g20470.t4	1048	3	NL
itf04g34230.t1	874	1	CNL
itf01g24580.t1	832	2	CNL
itf11g21040.t1	540	3	NL
itf15g22510.t1	583	2	CN
itf13g00800.t1	1108	1	NL
itf00g04820.t1	429	3	N
itf15g22520.t1	648	3	NL
itf07g22440.t1	735	2	CN
itf06g05600.t1	673	2	NL
itf13g02540.t1	869	1	CNL
itf09g25000.t1	920	3	CNL
itf13g03690.t1	1019	7	CNL
itf07g20970.t3	831	4	TNL

(*Continued*)

Table S3: *Continued*

Gene	No. of aa	No. of Exons	Type
itf14g01620.t1	1014	5	CNL
itf06g13610.t1	914	2	NL
itf13g03970.t1	892	2	NL
itf13g01670.t1	950	2	CN
itf06g13060.t4	794	2	NL
itf06g13070.t2	851	3	CNL
itf07g07860.t2	870	2	CN
itf15g10780.t1	2662	14	CNL
itf06g12210.t2	982	2	CNL
itf13g01010.t1	963	4	CN
itf15g18510.t1	669	2	CNL
itf13g01710.t2	938	4	CNL
itf13g01730.t1	909	4	CNL
itf09g25590.t1	926	1	CNL
itf01g20080.t1	914	1	CNL
itf04g01350.t1	843	8	CN
itf07g02380.t2	2208	25	TNL
itf13g00960.t1	1014	2	CNL
itf14g03860.t1	815	2	N
itf04g09550.t2	456	3	N
itf00g25720.t1	842	1	N
itf06g12180.t4	968	3	CNL
itf13g03710.t1	1013	1	CNL
itf04g01390.t2	1183	3	CNL
itf05g06500.t2	924	4	CNL
itf04g10000.t1	871	3	CN
itf13g21940.t1	733	3	NL
itf03g20500.t1	990	4	CNL
itf13g02100.t3	879	3	CNL
itf15g18430.t1	742	5	NL
itf00g25710.t1	455	2	N
itf03g21730.t1	916	2	CN
itf04g05490.t2	715	4	CN
itf13g21940.t4	725	3	NL
itf04g10060.t1	710	5	CNL
itf00g16810.t1	635	3	TNL
itf14g01620.t3	1014	5	CNL
itf07g07910.t1	887	3	CNL
itf03g21700.t1	950	3	N
itf08g14610.t1	1408	8	TNL

Table S3: *Continued*

Gene	No. of aa	No. of Exons	Type
itf03g20990.t2	1124	5	CNL
itf15g10820.t1	1027	3	CNL
itf03g12490.t1	880	1	CN
itf13g14140.t1	870	2	CNL
itf07g02520.t2	3140	22	TNL
itf03g21770.t1	779	3	CNL
itf07g07860.t1	870	3	CN
itf03g21720.t2	875	2	CNL
itf13g21950.t1	734	1	NL
itf13g21990.t2	726	1	NL
itf11g20300.t1	391	2	N
itf14g02060.t1	547	2	NL
itf04g05410.t2	918	2	CNL
itf13g02070.t1	956	3	CNL
itf14g02070.t1	782	1	NL
itf15g22460.t1	907	3	CNL
itf14g04720.t1	567	2	CN
itf06g12180.t8	968	1	CNL
itf08g09480.t2	1497	9	TNL
itf04g05390.t1	893	3	CNL
itf15g19840.t1	1223	5	CNL
itf00g11080.t3	876	6	NL
itf14g00250.t1	1013	2	NL
itf13g01750.t1	980	4	CNL
itf00g07890.t1	875	3	CNL
itf15g21480.t3	606	5	TN
itf00g10780.t2	1175	5	TNL
itf13g04870.t1	530	3	CN
itf07g07900.t1	847	2	CNL
itf11g05970.t1	1020	2	CNL
itf01g33990.t1	836	5	RNL
itf11g20610.t1	532	3	NL
itf13g14120.t2	723	4	NL
itf11g20430.t1	938	5	CNL
itf09g25000.t2	920	1	CNL
itf03g20560.t1	916	2	CNL
itf11g20490.t1	526	2	NL
itf00g08100.t1	1993	20	TNL
itf13g02410.t1	850	3	CNL
itf03g27310.t1	1067	3	NL

(*Continued*)

Table S3: *Continued*

Gene	No. of aa	No. of Exons	Type
itf15g00150.t1	473	1	CN
itf03g20470.t2	1048	4	NL
itf13g02090.t2	883	2	CNL
itf13g03690.t2	1019	2	CNL
itf14g01640.t3	691	2	NL
itf04g34220.t1	861	1	CNL
itf00g07890.t2	878	3	CNL
itf06g13060.t3	794	2	NL
itf09g24750.t1	903	3	CNL
itf05g06500.t1	917	4	CNL
itf07g09670.t1	238	1	N
itf13g02550.t1	1048	4	CN
itf06g12210.t1	976	5	CNL
itf07g14280.t1	930	6	N
itf01g10090.t1	504	2	NL
itf14g03870.t1	772	2	NL
itf04g05380.t1	899	3	CNL
itf00g00070.t1	987	4	CNL
itf13g01030.t1	1276	3	CNL
itf06g20590.t1	911	2	CNL
itf15g10770.t1	1311	6	CNL
itf11g20440.t2	918	3	CNL
itf04g13450.t1	856	1	CNL
itf07g02310.t1	1800	17	TNL
itf03g22030.t1	882	2	CN
itf15g22060.t1	658	5	N
itf09g24780.t2	892	3	CNL
itf01g18600.t1	987	3	TN
itf15g19820.t1	834	6	NL
itf11g20510.t1	917	3	CNL
itf15g10800.t1	1402	8	CNL
itf08g17370.t1	661	2	CN
itf04g05490.t1	933	3	CNL
itf11g20320.t1	519	2	N
itf14g04500.t1	887	3	CNL
itf14g01620.t2	1014	4	CNL
itf11g20670.t1	913	3	CNL
itf15g10800.t2	1405	7	CNL
itf14g02040.t1	1663	3	NL
itf13g21940.t5	733	3	NL

Table S3: *Continued*

Gene	No. of aa	No. of Exons	Type
itf04g10190.t1	726	3	NL
itf07g02310.t4	1393	7	TNL
itf08g09480.t4	1349	8	NL
itf06g12280.t1	951	1	NL
itf13g21960.t1	729	1	NL
itf00g08650.t1	392	1	N
itf03g27300.t1	620	1	NL
itf05g06510.t2	948	4	CNL
itf00g20060.t1	773	5	NL
itf06g10110.t2	905	3	NL
itf15g10700.t1	1825	6	CNL
itf11g20040.t3	1006	2	CNL
itf03g27550.t1	821	2	NL
itf11g20590.t1	766	3	N
itf04g01370.t1	625	6	N
itf06g12180.t2	987	4	CNL
itf13g01680.t1	985	4	CNL
itf06g13050.t1	811	3	NL
itf15g04690.t1	1137	3	NL
itf04g10000.t3	871	2	CN
itf06g13100.t2	874	3	CNL
itf09g24960.t1	926	1	CNL
itf07g20970.t1	922	5	TNL
itf01g08960.t1	651	3	N
itf14g02030.t1	1749	4	NL
itf11g20430.t2	938	4	CNL
itf03g20740.t1	892	3	CNL
itf06g10010.t1	847	1	NL
itf03g23010.t1	845	4	CNL
itf15g18540.t1	359	1	N
itf04g04430.t1	973	1	CNL
itf13g21940.t6	733	2	NL
itf06g10580.t1	850	1	NL
itf15g22420.t1	455	3	N
itf07g02370.t1	1736	16	TNL
itf11g20430.t3	938	3	CNL
itf13g00810.t1	632	3	N
itf00g08680.t2	376	3	N
itf03g20730.t1	887	3	CNL
itf07g22460.t1	495	1	N

(*Continued*)

Table S3: *Continued*

Gene	No. of aa	No. of Exons	Type
itf03g28470.t1	855	2	CNL
itf01g21600.t1	588	2	NL
itf13g21940.t2	731	2	NL
itf12g13120.t2	735	2	NL
itf15g18440.t1	834	5	NL
itf11g13930.t1	635	3	NL
itf13g00840.t1	646	2	N
itf13g01450.t1	798	7	CN
itf05g11580.t1	478	1	CN
itf00g30960.t1	991	3	CNL
itf07g02310.t2	1815	15	TNL
itf12g21480.t1	756	4	TN
itf10g07890.t1	676	5	N
itf15g18560.t1	856	5	NL
itf15g10700.t2	1825	6	CNL
itf02g11630.t1	877	3	CNL
itf14g01640.t1	880	3	CNL
itf04g08640.t1	784	1	NL
itf06g13100.t1	874	3	CNL
itf06g09970.t1	516	2	N
itf13g14120.t1	756	3	NL
itf11g20080.t1	421	3	N
itf14g03840.t1	766	2	NL
itf01g10890.t1	606	2	CNL
itf09g24980.t1	927	1	CNL
itf14g04500.t2	887	2	CNL
itf14g04520.t1	688	3	CNL
itf13g02100.t2	889	4	CNL
itf04g09990.t2	882	2	CN
itf14g02110.t1	886	4	CN
itf04g07270.t1	1215	2	NL
itf13g12870.t1	888	3	CNL
itf04g09260.t1	851	4	CN
itf00g29730.t1	765	1	NL
itf08g14640.t1	2618	28	TNL
itf04g10000.t2	871	3	CN
itf11g20290.t1	442	3	N
itf03g22050.t1	877	1	CN
itf04g34280.t1	1273	1	CNL
itf04g09550.t1	642	4	TN

Table S3: *Continued*

Gene	No. of aa	No. of Exons	Type
itf00g01120.t1	829	3	NL
itf11g20660.t1	712	3	NL
itf00g24800.t1	875	2	CNL
itf07g02380.t1	2193	25	TNL
itf02g01860.t1	760	3	CNL
itf13g03950.t1	1014	5	CNL
itf14g00240.t1	890	1	CNL
itf13g01700.t1	1143	3	CN
itf03g20560.t3	916	2	CNL
itf11g20280.t1	484	3	N
itf14g01630.t1	723	3	NL
itf14g02100.t1	1657	4	CNL
itf14g02090.t1	888	3	CNL
itf07g22420.t1	778	3	CN
itf11g21840.t1	400	3	NL
itf04g04440.t1	931	1	CNL
itf09g16380.t2	876	4	TNL
itf07g22430.t1	437	1	CN
itf04g10180.t1	886	2	CNL
itf01g19840.t1	719	2	NL
itf11g20480.t1	455	3	N
itf15g22490.t1	528	2	CN
itf07g02370.t2	1610	15	TNL
itf12g05370.t1	828	5	RNL
itf03g21720.t1	936	3	CNL
itf11g20580.t1	472	3	NL
itf06g10110.t1	905	3	NL
itf04g08850.t1	699	3	CN
itf14g01400.t2	708	2	NL
itf13g01720.t1	1015	1	CNL
itf13g21940.t3	679	4	NL
itf11g18990.t1	842	3	CNL
itf06g12270.t1	973	1	CNL
itf09g21670.t1	670	3	NL
itf03g20470.t3	1048	4	NL
itf00g32110.t1	511	2	CN
itf07g16420.t8	1562	15	NL
itf00g11230.t1	1678	6	NL
itf07g07850.t1	867	3	CNL
itf10g12820.t1	664	2	NL

(*Continued*)

Table S3: *Continued*

Gene	No. of aa	No. of Exons	Type
itf00g30960.t3	991	2	CNL
itf14g02120.t1	897	2	CNL
itf15g22190.t1	873	3	CNL
itf03g20730.t2	706	2	NL
itf15g10880.t1	2059	9	CNL
itf15g22050.t1	726	4	N
itf00g08120.t1	1004	6	TNL
itf03g20950.t1	637	4	NL
itf07g02520.t1	3145	22	TNL
itf00g16800.t2	573	4	TN
itf09g24820.t1	780	3	CNL
itf06g13060.t1	796	3	NL
itf11g20650.t1	382	2	N
itf09g24990.t1	922	1	CNL
itf09g24780.t1	892	3	CNL
itf08g12730.t5	993	3	NL
itf03g20560.t2	916	1	CNL
itf10g05780.t1	1236	5	TNL
itf09g24740.t1	714	1	NL
itf07g02380.t3	2179	24	TNL
itf13g03750.t1	1272	4	CNL
itf11g20440.t1	918	3	CNL
itf13g01480.t1	899	4	CN
itf11g13960.t1	529	2	N
itf06g10030.t2	985	2	NL
itf06g03570.t1	867	1	NL
itf06g12180.t7	968	2	CNL
itf14g06890.t1	897	1	CN
itf15g19820.t2	814	4	NL
itf09g17780.t1	498	6	N
itf11g20050.t1	577	2	NL
itf06g13070.t3	851	2	CNL
itf14g01400.t3	891	2	CNL
itf11g20040.t1	997	3	CNL
itf09g24770.t1	940	4	CNL
itf00g19420.t1	897	4	TNL
itf01g19690.t1	905	2	CNL
itf11g20570.t1	925	3	CN
itf01g20130.t1	931	1	CNL
itf11g20040.t2	997	3	CNL

Table S3: *Continued*

Gene	No. of aa	No. of Exons	Type
itf00g30960.t2	991	3	CNL
itf03g22060.t1	815	1	N
itf00g08690.t1	729	2	NL
itf07g16420.t7	1673	17	NL
itf00g08120.t2	966	5	TNL
itf06g12180.t1	987	5	CNL
itf12g13120.t1	735	4	NL
itf15g21980.t1	587	3	CN
itf06g20590.t3	911	1	CNL
itf08g09480.t3	1245	9	TNL
itf07g02310.t3	1753	14	TNL
itf06g12290.t1	994	3	CNL
itf06g06580.t1	845	3	NL
itf13g01470.t1	1077	2	CNL
itf07g20970.t2	780	5	TNL
itf09g24790.t1	911	1	CNL
itf00g16800.t1	642	5	TNL
itf06g13100.t3	874	2	CNL
itf00g11230.t2	1678	6	NL
itf07g07910.t2	709	2	NL
itf04g05400.t1	634	3	CN
itf08g12730.t1	1367	5	TNL
itf15g22470.t1	495	3	NL
itf15g17060.t2	607	3	TN
itf13g01680.t2	985	4	CNL
itf15g22210.t2	723	5	N
itf09g25610.t1	930	1	CNL
itf09g24730.t1	886	1	CNL
itf04g10200.t1	528	2	CN
itf13g01750.t2	1086	3	CN
itf15g22000.t1	652	3	CN
itf03g21750.t1	731	3	CN
itf03g21020.t1	1058	4	CNL
itf03g20760.t1	902	2	CN
itf14g02290.t1	575	3	CN
itf06g13090.t1	910	3	CNL
itf13g01710.t1	540	4	CN
itf07g22370.t1	559	1	CN
itf07g07830.t1	867	3	CN
itf00g24790.t1	875	3	CNL

(*Continued*)

Table S3: *Continued*

Gene	No. of aa	No. of Exons	Type
itf00g11080.t2	1224	7	TNL
itf02g11640.t1	878	3	CNL
itf00g10780.t1	1000	4	TNL
itf11g21790.t1	388	2	N
itf03g22040.t1	808	1	NL
itf15g19850.t1	817	6	NL
itf06g13070.t1	807	4	CNL
itf04g01390.t1	1318	4	CNL
itf13g03980.t1	1078	3	CNL
itf08g12730.t4	1147	4	NL
itf07g16420.t6	1664	16	NL
itf15g22550.t1	389	2	N
itf07g02310.t5	1645	14	TNL
itf14g01400.t1	898	3	CNL
itf09g07950.t1	944	3	CNL
itf09g24850.t1	897	1	CNL
itf00g29740.t1	765	1	NL
itf15g22210.t1	1252	7	CN
itf15g21910.t1	622	3	NL
itf13g02090.t1	883	3	CNL
itf06g12180.t6	987	2	CNL

Table S3: *Continued*

Gene	No. of aa	No. of Exons	Type
itf06g12250.t1	886	4	CNL
itf07g16420.t2	2098	25	NL
itf00g08630.t1	725	2	NL
itf04g01400.t1	505	4	N
itf15g22020.t1	677	3	NL
itf13g02100.t1	889	4	CNL
itf08g12730.t3	1213	4	TNL
itf06g05520.t1	630	2	NL
itf07g16830.t1	748	3	N
itf04g08290.t1	977	3	CNL
itf03g20470.t1	1048	4	NL
itf00g20300.t1	426	2	N
itf14g01640.t2	880	2	CNL
itf11g20070.t1	385	2	N
itf14g00270.t1	892	2	CNL
itf06g12150.t1	980	1	CNL
itf03g22010.t1	721	1	NL
itf04g09990.t1	882	3	CN
itf01g20090.t1	941	1	CNL
itf06g13060.t2	796	3	NL

Table S4: Duplication analysis of *I. trifida* NBS encoding genes

Type	NO. of genes	Average NO. of aa	Average NO. of exons
N	46	547.61	2.85
TN	8	644.88	3.38
CN	58	737.76	2.76
RNL	2	832.00	5.00
NL	118	870.23	3.82
CNL	175	976.23	2.99
TNL	35	1473.71	10.80
All	442	904.78	3.80

Table S5: The number of aa and exons of different types of *I.trifida* NBS genes

Gene pairs	Duplication type	K_a/K_s	*T* (Mya)
itf01g20080.t1_itf01g20090.t1	Tandem	0.91	50.11
itf03g20980.t1_itf08g14640.t1	Segmental replication	0.92	82.46
itf03g21730.t1_itf03g21720.t2	Tandem	1.06	48.84
itf03g21730.t1_itf04g09550.t1	Segmental replication	0.78	94.09
itf03g22030.t1_itf03g22010.t1	Tandem	0.93	81.45
itf03g22050.t1_itf03g22040.t1	Tandem	1.01	76.48
itf03g22050.t1_itf03g22060.t1	Tandem	0.94	80.96
itf04g01390.t4_itf04g01400.t1	Tandem	0.91	83.12
itf04g04430.t1_itf04g04440.t1	Tandem	0.99	77.39
itf04g04450.t1_itf04g04440.t1	Tandem	0.92	82.13
itf04g05390.t1_itf04g05400.t1	Tandem	0.99	77.26
itf04g06680.t1_itf15g19820.t1	Segmental replication	0.98	78.24
itf04g13460.t1_itf04g13450.t1	Tandem	0.99	77.54
itf04g13460.t1_itf04g13470.t1	Tandem	1.01	76.43
itf06g10010.t1_itf06g09970.t1	Tandem	1.05	74.08
itf06g13060.t3_itf06g13060.t2	Tandem	0.59	1.37
itf06g13060.t4_itf06g13070.t1	Tandem	1.04	74.34
itf06g13100.t1_itf06g13090.t1	Tandem	1.18	67.58
itf06g13610.t1_itf01g19840.t1	Segmental replication	0.84	87.83
itf06g13610.t1_itf04g08850.t1	Segmental replication	0.89	84.41
itf07g02310.t2_itf07g02310.t3	Tandem	0.94	81.03
itf07g02310.t3_itf07g02310.t5	Tandem	0.91	83.23
itf07g02380.t2_itf07g02380.t1	Tandem	1.06	73.27
itf07g02380.t2_itf07g02380.t3	Tandem	0.95	80.13
itf07g16420.t1_itf04g08850.t1	Segmental replication	1.14	69.02
itf07g16420.t1_itf04g09950.t1	Segmental replication	1.09	71.67
itf07g16420.t7_itf07g16420.t6	Tandem	36.24	0.00
itf07g16420.t8_itf07g16420.t6	Tandem	1.10	71.39
itf07g20970.t3_itf07g20970.t2	Tandem	1.10	71.13
itf07g22440.t1_itf07g22460.t1	Tandem	0.92	82.31
itf07g22470.t1_itf07g22460.t1	Tandem	0.74	97.07
itf08g09480.t4_itf08g09480.t3	Tandem	0.91	83.13
itf08g12730.t4_itf08g12730.t3	Tandem	1.05	73.97
itf09g24950.t1_itf09g24960.t1	Tandem	0.72	12.16
itf09g24960.t1_itf09g24980.t1	Tandem	1.08	9.48
itf09g24980.t1_itf09g24990.t1	Tandem	1.33	8.34
itf09g25000.t1_itf09g24990.t1	Tandem	0.95	6.31
itf11g20300.t1_itf11g20290.t1	Tandem	1.05	74.11
itf11g20450.t1_itf11g20440.t2	Tandem	0.85	87.31
itf11g20470.t1_itf11g20480.t1	Tandem	0.88	85.44
itf11g20590.t1_itf11g20580.t1	Tandem	1.18	67.47
itf11g20660.t1_itf11g20650.t1	Tandem	1.18	67.23
itf12g13120.t3_itf12g13120.t2	Tandem	0.28	0.19
itf13g00960.t1_itf03g20730.t1	Segmental replication	0.92	82.64
itf13g00960.t1_itf03g21700.t1	Segmental replication	1.02	75.45
itf13g00960.t1_itf04g09260.t1	Segmental replication	1.01	76.42
itf13g01010.t1_itf03g20950.t1	Segmental replication	1.07	72.81
itf13g01010.t1_itf03g22010.t1	Segmental replication	0.91	82.58
itf13g01670.t1_itf03g20950.t1	Segmental replication	0.84	87.83
itf13g01670.t1_itf13g01680.t1	Tandem	0.86	86.22
itf13g02070.t1_itf03g20730.t1	Segmental replication	1.04	74.68
itf13g02070.t1_itf04g10180.t1	Segmental replication	0.79	91.94
itf13g02070.t1_itf15g19820.t1	Segmental replication	0.90	83.70
itf13g02100.t3_itf13g02100.t2	Tandem	9.18	0.02
itf13g02100.t4_itf13g02100.t3	Tandem	0.89	84.11
itf13g02410.t1_itf04g09260.t1	Segmental replication	1.13	69.55
itf13g02540.t1_itf13g02550.t1	Tandem	1.14	69.45
itf13g21940.t2_itf13g21940.t3	Tandem	0.77	94.29
itf13g21940.t4_itf13g21940.t3	Tandem	0.76	95.28
itf14g01400.t2_itf14g01400.t1	Tandem	1.08	72.30
itf14g01400.t2_itf14g01400.t3	Tandem	1.07	72.73

(*Continued*)

Table S5: *Continued*

Gene pairs	Duplication type	K_a/K_s	T (Mya)
itf14g01640.t3_itf14g01640.t2	Tandem	0.92	82.13
itf14g02050.t1_itf14g02060.t1	Tandem	1.00	76.80
itf14g02060.t1_itf14g02070.t1	Tandem	1.29	62.89
itf14g02080.t1_itf14g02070.t1	Tandem	0.83	88.63
itf14g02110.t1_itf14g02120.t1	Tandem	0.70	62.83
itf14g03860.t1_itf14g03870.t1	Tandem	0.98	78.49
itf15g10800.t1_itf15g10800.t2	Tandem	0.00	0.09
itf15g18510.t1_itf15g18440.t1	Tandem	0.89	84.63
itf15g19820.t1_itf15g19820.t2	Tandem	0.80	91.40
itf15g22210.t2_itf15g22210.t1	Tandem	0.90	83.23
itf15g22510.t1_itf15g22520.t1	Tandem	0.98	78.07
